# Nanomaterials that Aid in the Diagnosis and Treatment of Alzheimer's Disease, Resolving Blood–Brain Barrier Crossing Ability

**DOI:** 10.1002/advs.202403473

**Published:** 2024-08-05

**Authors:** Qingting Song, Junyou Li, Ting Li, Hung‐Wing Li

**Affiliations:** ^1^ Department of Chemistry The Chinese University of Hong Kong Hong Kong China

**Keywords:** Alzheimer's disease, blood–brain barrier, diagnosis and treatment, nanomaterials

## Abstract

As a form of dementia, Alzheimer's disease (AD) suffers from no efficacious cure, yet AD treatment is still imperative, as it ameliorates the symptoms or prevents it from deteriorating or maintains the current status to the longest extent. The human brain is the most sensitive and complex organ in the body, which is protected by the blood–brain barrier (BBB). This yet induces the difficulty in curing AD as the drugs or nanomaterials that are much inhibited from reaching the lesion site. Thus, BBB crossing capability of drug delivery system remains a significant challenge in the development of neurological therapeutics. Fortunately, nano‐enabled delivery systems possess promising potential to achieve multifunctional diagnostics/therapeutics against various targets of AD owing to their intriguing advantages of nanocarriers, including easy multifunctionalization on surfaces, high surface‐to‐volume ratio with large payloads, and potential ability to cross the BBB, making them capable of conquering the limitations of conventional drug candidates. This review, which focuses on the BBB crossing ability of the multifunctional nanomaterials in AD diagnosis and treatment, will provide an insightful vision that is conducive to the development of AD‐related nanomaterials.

## Graphical abstract

Strategic design of different formulations of nanomaterials‐mediated molecule delivery systems with enhanced blood–brain barrier (BBB) crossing ability, as well as multiplexed mechanism of Alzheimer's disease (AD) for AD therapy and diagnosis is presented.

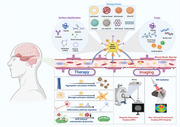

## 1 Introduction

**Figure 1:**
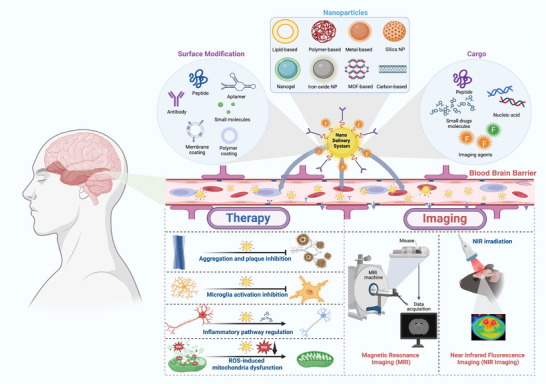
Nanomaterials‐mediated drug delivery with enhanced BBB crossing ability for AD treatment and diagnosis. Created with BioRender.com.

**Figure 2:**
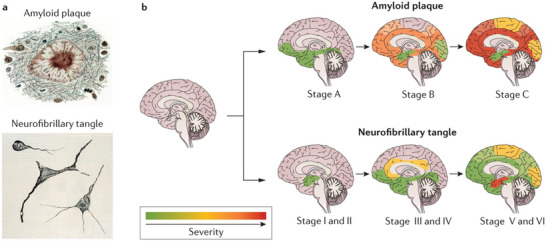
a) Amyloid plaques and neurofibrillary tangles spread through the brain as the disease progresses. b) Amyloid‐β (Aβ) deposition precedes neurofibrillary and neurotic changes with an apparent origin in the frontal and temporal lobes, hippocampus, and limbic system (top row). The neurofibrillary tangles and neurotic degeneration start in the medial temporal lobes and hippocampus, progressively spreading to other areas (bottom row). Reproduced with permission.^[^ ^1^ ^]^ Copyright 2015, Springer Nature.

In this review, we focus on the blood‐brain‐barrier crossing ability of the multifunctional nanomaterials in AD diagnosis and treatment (as illustrated in **Figure** **1**), and provide an insightful vision that is conducive to the development of AD‐related nanomaterials. Alzheimer's disease (AD) is known to be a progressive, unremitting, neurodegenerative disorder that affects wide areas of the cerebral cortex and hippocampus. Abnormalities are usually first detected in the brain tissue involving the frontal and temporal lobes, then slowly progressing to other areas of the neocortex at rates that vary considerably between individuals, illustrated in **Figure** **2**.^[^ ^1^ ^]^

According to the latest report from Alzheimer's Association, there are ≈6.9 million people aged 65 and older living with AD in the United States alone.^[^ ^2^ ^]^ The WHO estimates that more than 55 million individuals worldwide are affected by dementia, with AD being the most prevalent form and potentially accounting for 60–70% of cases.^[^ ^3^, ^4^ ^]^ As a disease impairing memory and cognitive ability,^[^ ^5^ ^]^ AD progresses slowly and leaves end‐stage patients bedridden, incontinent, and dependent on custodial care, causing significant burdens both financially and mentally to both the individuals and their families and care providers. AD also poses a great challenge to the aging society. As global life expectancy increases, the number of people living with AD is projected to reach 87 million by 2050, according to the WHO.^[^ ^6^ ^]^ The costs associated with dementia are also expected to more than double from US$1.3 trillion per year in 2019 to 2.8 trillion dollars by 2030. Moreover, these debilitating and financially devastating diseases are expected to increase into the middle of the century.^[^ ^7^ ^]^

While remarkable advances in recent years in the diagnosis and treatment of dementia have been witnessed,^[^ ^8^ ^]^ we are still far from having a curing solution and even further away from forming healthcare systems capable of disseminating a future remedy worldwide.

### 1.1 Pathogenesis and Therapeutic Strategies of AD

**Figure 3:**
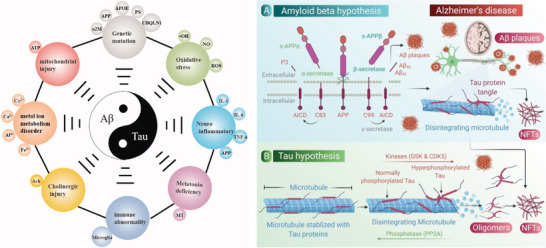
Schematic illustration of the diversity of AD pathogenesis (left). Reproduced with permission.^[^ ^14^ ^]^ Copyright 2021, Taylor & Francis. The Aβ and Tau hypotheses in AD (right). Reproduced under the terms of the Creative Commons Attribution‐Noncommercial‐Noderivs 4.0 International Public License.^[^ ^23^ ^]^ Copyright 2022, Bhardwaj et al.

AD is a rather complex neurodegenerative disease that is attributed to a combination of multiple factors.^[^ ^9^ ^]^ Among the various pathological pathways involved, synaptic dysfunctions, such as synapse loss and deficits in synaptic plasticity, are strongly associated with cognitive decline.^[^ ^10^, ^11^ ^]^ Neurotransmitter deficiencies also contribute to the diverse neurodegenerative symptoms observed in AD, including cholinergic and glutamatergic deficits for cognitive decline,^[^ ^12^ ^]^ excitatory and inhibitory neurotransmission dyshomeostasis for synaptic plasticity deficits and epileptiform symptoms, and monoamine neurotransmission for neuropsychiatric symptoms.^[^ ^13^ ^]^ Some approved drugs for ameliorating AD symptoms are predicated on this mechanism, such as donepezil, rivastigmine, and galantamine, functioning as acetylcholinesterase inhibitors (AChEI) to impede the breakdown of acetylcholine (ACh) by acetylcholinesterase (AChE), thus increasing concentration of ACh and fostering enhanced interneuronal communication. Apart from these, four other important hypotheses for AD pathogenesis are proposed: Aβ protein cascade theory, Tau protein hyperphosphorylation theory, mitochondrial dysfunction and oxidative stress theory, and neuroinflammatory response,^[^ ^14^, ^15^ ^]^ among which the amyloid cascade hypothesis and Tau hypothesis are the most popular two (**Figure** **3**). The Aβ cascade theory suggests that the accumulation of neurotoxic Aβ protein, derived from the breakdown of amyloid precursor protein (APP), results in the formation of dense fibrous plaques in the brain. In pathological conditions, APP is hydrolysed by β‐secretase or γ‐secretase to generate Aβ_40_ (with 40 amino acids) or Aβ_42_ protein (with 42 amino acids), respectively, with three forms of Aβ species: monomers, oligomers, and fibrils, where oligomeric form exhibits a higher toxicity and detrimental impact on neurons than monomers and fibrils. They cause oxidative damage to neurons, dysfunction of synapses, neuroinflammation, and apoptosis of nerve cells, leading to further deterioration and, ultimately, the development of AD.^[^ ^16^, ^17^, ^18^ ^]^ The Tau protein hyperphosphorylation hypothesis proposes that abnormal phosphorylation of Tau proteins leads to the formation of neurofibrillary tangles (NFTs), which disrupts the normal structure and function of neurons. These NFTs are made up of paired helical filaments that are assembled by Tau, a microtubule‐associated protein. In AD, the dissociation of Tau protein destabilizes microtubules, leading to the deterioration of neural functions. While Tau phosphorylation is a normal process in healthy conditions, pathological conditions such as Aβ toxicity, neuroinflammation, and other stress factors can cause aberrant Tau phosphorylation. This imbalance in Tau kinase and phosphatase activities contributes to the aggregation of Tau, ultimately leading to the degeneration and death of nerve cells in AD.^[^ ^19^, ^20^ ^]^ It is suggested that mitochondrial dysfunction caused by oxidative stress plays a significant role in AD pathogenesis. Oxidative stress arises from an imbalance between reactive oxygen species (ROS) production and antioxidant defence, leading to the compromise of mitochondria by free radicals. Mitochondrial dysfunction induces the production of Aβ and elevates Tau phosphorylation. Consequently, heightened Aβ levels may enhance ROS production, alter oxidative phosphorylation, and engage with mitochondrial dynamics and matrix proteins to worsen mitochondrial impairment, establishing a “vicious cycle” culminating in neuronal damage.^[^ ^21^ ^]^ Last but not least, chronic inflammation mediated by microglia in the brain is suggested to contribute to the progression of AD. Microglia are specialized immune cells in the CNS that can activate and release inflammatory factors. Excessively activated microglia in the brain of AD patients release a large number of inflammatory factors and neurotoxic substances, which lead to harmful effects, such as increased inflammation, acceleration of Aβ deposition, promotion of neuronal damage, and aggravation of AD. Inflammatory processes involve the activation of immune cells and the release of proinflammatory molecules, leading to neuronal dysfunction and cell death.^[^ ^22^ ^]^

To intervene in AD, a variety of approaches have been explored including pharmacological interventions, gene therapy, and nanotechnology‐based drug delivery systems. Given the mentioned hypotheses for AD, researchers have adopted corresponding strategies to regulate the occurrence and development of AD. These strategies are summarized as follows: 1) by reducing the production of Aβ, accelerating the clearance of Aβ, and attenuating the deposition of amyloid plaques; 2) by controlling the stability of microtubules, reducing the expression of Tau protein, inhibiting abnormal phosphorylation of Tau protein, and obtain normal function of Tau protein; 3) by inhibiting microglia activation to improve synaptic plasticity and neural regeneration, or directly augmenting various sorts of neurotransmitter deficiency; 4) by taking anti‐inflammatory drugs to regulate inflammation pathway; 5) by reducing oxidative stress and regulating homeostasis to maintain the normal function of mitochondria.^[^ ^14^ ^]^ Given the diversity and complexity of AD pathogenesis, therapeutic strategies now focus on multitarget therapies to address the multifaceted nature of AD.^[^ ^9^ ^]^

### 1.2 Diagnosis and Treatment of AD

Currently, there is no cure for AD but relieving the symptoms only. The optimal time window for AD treatment is still indefinable due to the lack of confirmative diagnostics. The up‐to‐date diagnostic approaches for AD primarily rely on the detection of pathological factors as biomarkers, including in vitro quantitative analysis of cerebrospinal fluid (CSF) or blood and in vivo brain neuroimaging of Aβ or p‐Tau.^[^ ^24^ ^]^ Imaging technologies such as positron emission tomography (PET), single‐photon emission computed tomography, magnetic resonance imaging (MRI), and near‐infrared (NIR)‐fluorescence imaging can detect AD at the research level.^[^ ^25^, ^26^, ^27^ ^]^ NIR fluorescence imaging is an emerging tool for the early detection of AD pathological features in animal models for understanding the disease mechanism.^[^ ^28^, ^29^, ^30^, ^31^, ^32^, ^33^ ^]^ PET imaging can clinically detect Aβ and Tau pathology with a radiotracer. However, it is costly and has limited availability for primary patient screening.^[^ ^34^ ^]^ MRI is a nonionizing imaging technique with high permeability, presenting the anatomical details and pathological information of organs and soft tissues at high resolution. Despite the wide use of MRI as a clinical diagnostic tool, there are still no clinically approved MRI contrast agents that can be applied for AD imaging.^[^ ^35^, ^36^ ^]^ The advancement of nanotechnology, however, offers vast prospects for its applications in bioimaging for disease detection for their unique physicochemical features.^[^ ^37^ ^]^ For instance, superparamagnetic iron oxide nanoparticles (SPIO NPs) are renowned “negative” contrast agents for T_2_‐weighted MRI in various diseases.

For the treatment of AD, the accessible anti‐AD drugs can only alleviate clinical symptoms but not reverse or halt the progression of the disease, yet a total of eight FDA‐approved drugs are made available as of current. Five of these drugs, donepezil, rivastigmine, galantamine, memantine, and memantine combined with donepezil, are aimed at treating cognitive symptoms. Another one, brexpiprazole, has been approved to treat agitation that can occur in AD. Two additional drugs, aducanumab and lecanemab, change the underlying biology of AD and slow cognitive and functional decline in some individuals. Among them, donepezil, rivastigmine, and galantamine are AChEI, which do not affect the underlying brain changes that cause AD, nor do they slow or stop the course of the disease. Memantine treats symptoms by increasing neurotransmitters in the brain and can protect the brain from excessive levels of a neurotransmitter called glutamate, which overstimulates and damages neurons. Brexpiprazole works through its effects on dopamine and serotonin receptors in the brain. Aducanumab and lecanemab can delay disease progression by helping remove plaques and Aβ protofibrils, but they may cause significant side effects, such as perivascular edema and hemorrhages. The approval of a third such drug, donanemab, has been delayed by the FDA considering its safety and efficacy.^[^ ^2^ ^]^ However, all the drugs currently approved for treating AD are available as oral formulations, except Rivastigmine.^[^ ^38^ ^]^ Since these drugs need to reach the CNS to control the progression of the disease or its symptoms, a much higher dose needs to be consumed because a large fraction of the drug could be lost in the gastrointestinal tract or metabolized in the hepatic region.^[^ ^39^ ^]^ Furthermore, these drugs also bind to serum albumin in the bloodstream to sustain a decent half‐life before it finally reaches the BBB.^[^ ^40^ ^]^ Consuming high dosages leads to side effects like nausea and diarrhea, and drugs’ compatibility is sacrificed. Moreover, there are other significant limitations to the targeted delivery of these drugs to the CNS, including lower bioavailability, solubility, and reduced efficacy due to the BBB. Thankfully, recent advancements in nanotechnology show promise in overcoming these limitations, especially for efficient brain drug delivery crossing the BBB.^[^ ^37^, ^41^, ^42^, ^43^, ^44^, ^45^ ^]^

In short, the primary prerequisite and challenge for both imaging‐based diagnosis and treatment for AD, as well as other brain diseases, is the need to deliver imaging agents or therapeutic agents across the BBB.^[^ ^46^, ^47^ ^]^ Nanotechnological advancements in recent years have provided us with the option of nanomaterials that can aid in overcoming these hurdles in drug delivery,^[^ ^48^ ^]^ especially in ameliorating side effects by sustained drug release for reducing the dosage,^[^ ^49^ ^]^ enhancing bioavailability and pharmaceutical kinetics,^[^ ^50^ ^]^ and improving BBB crossing ability for targeted delivery of the drug.^[^ ^51^ ^]^ Thus, nanomaterials are promising and have been gaining soaring attention and devotion from researchers to cross BBB efficiently and realize efficacious AD diagnosis and treatment. In this review, we categorize different sorts of nanomaterials and discuss how they are rendered capable of crossing BBB.

## 2 The Difficulty of Transporting Drugs across the BBB

### 2.1 Physiology of BBB

**Figure 4:**
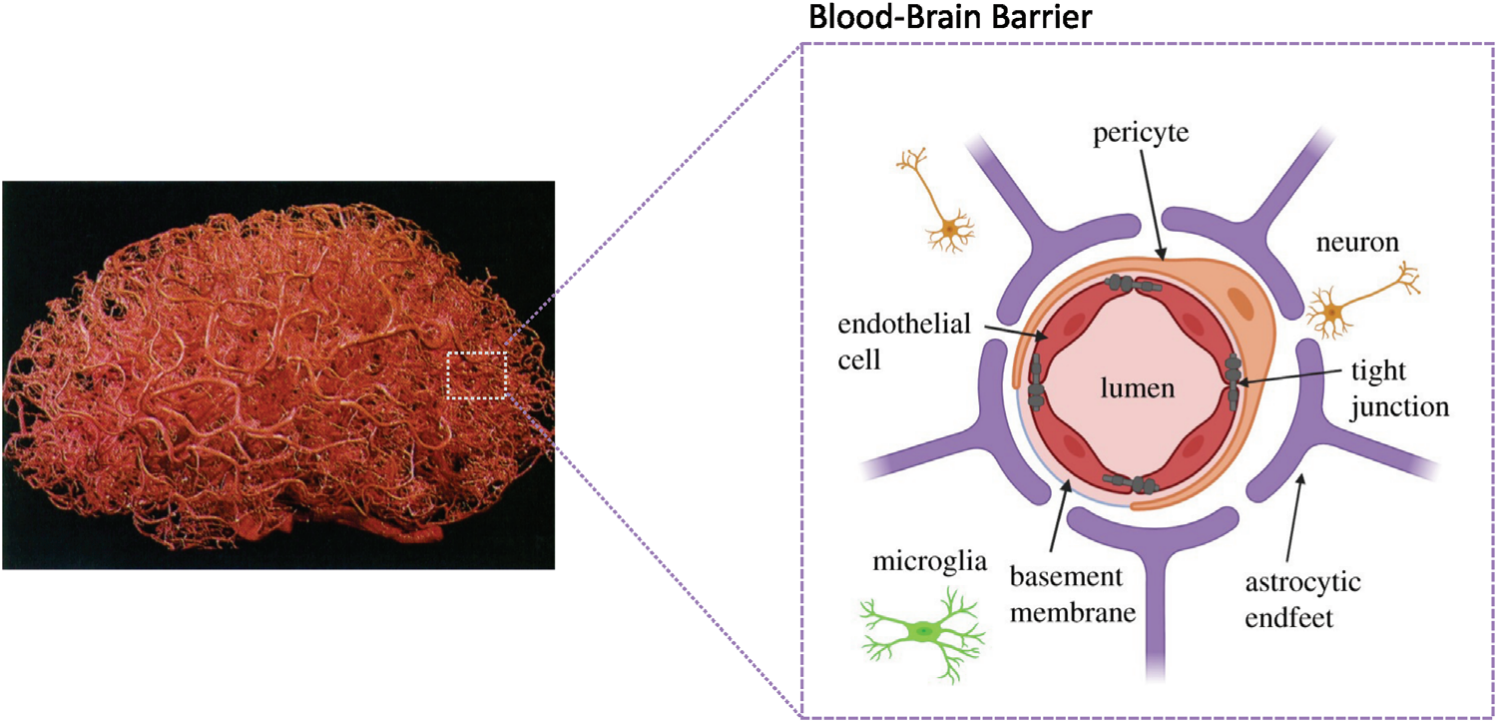
Blood vessels in the human brain (left). Reproduced with permission.^[^ ^55^ ^]^ Copyright 1998, Wolters Kluwer Health. Diagram of BBB structure (right). Reproduced under the terms of the Creative Commons Attribution 4.0 International Public License.^[^ ^56^ ^]^ Copyright 2021, Aryal et al.

The therapeutic targets aiming to treat AD should function mainly inside the brain. However, the presence of BBB poses the most significant challenge to drug delivery into the brain, which is a complex interface that is in intimate communication with the rest of the CNS and influenced by peripheral tissues.^[^ ^52^ ^]^ The primary function of the BBB is to protect the brain from potentially harmful substances in the bloodstream, but this feature also hinders most drugs from accessing the brain. It prevents most large molecules and ≈98% of small molecules from entering the brain. As illustrated in **Figure** **4**, BBB consists of brain capillary endothelial cells (ECs) joined by tight junctions, basement membrane, pericytes, and astrocyte endfeet surrounding the capillaries. It has a surface area of ≈20 m^2^, and the length of human brain capillaries is ≈400 miles. The ECs layer is lined with brush‐like glycoproteins (glycocalyx) and tightly tethered via tight junction protein connections. A continuous endothelial basement membrane, pericytes, and the astrocyte end‐feet cover the ECs.^[^ ^47^, ^52^, ^53^ ^]^ Because the neural cell is close to a capillary, by no further than 25 µm, passing the BBB for drug delivery is a favorable route compared to other comparatively longer by‐passing routes.^[^ ^54^ ^]^ This urges researchers to develop effective strategies to manipulate BBB permeability and, hence, targeted delivery systems.

### 2.2 In Vitro Models of BBB

The most important criteria for assessing NPs are their ability to accurately evaluate transfer across the BBB and subsequent release of the therapeutic payload in the targeted cells. To study the BBB penetrability of newly developed materials, scientists have developed in vitro BBB models, aiming to mimic the physiological and functional characteristics of the BBB.^[^ ^57^ ^]^ Most initial work on NPs is conducted via in vitro BBB models before in vivo testing.^[^ ^57^ ^]^

**Figure 5:**
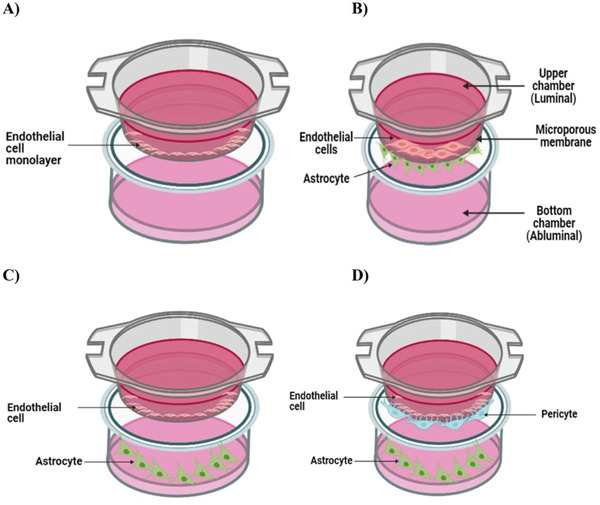
Schematic representation of in vitro transwell BBB models. A) Monoculture of ECs, B) contact and C) noncontact coculture of ECs and astrocytes, D) triple‐coculture system of ECs, pericytes, and astrocytes. Reproduced with permission.^[^ ^58^ ^]^ Copyright 2022, Springer Nature.

The most common in vitro model of the BBB is the transwell BBB model, where a monolayer of ECs grows on one side of a porous membrane submerged in the culture medium (**Figure** **5**A). This allows researchers to simulate and measure the ability of substances to cross from the upper side (representing blood) to another side (representing the brain). The simplicity of in vitro transwell BBB models lends itself to high‐throughput screening of NPs and facilitates rapid optimization of experimental conditions.^[^ ^59^ ^]^ However, to more closely mimic the in vivo characteristics of BBB, a second cell type, such as astrocytes, can be cocultured in the lower compartment either in contact (grown on the underside of the membrane) or noncontact (grown at the bottom of the lower compartment), as represented in Figure 5B,C. The simplicity of in vitro transwell BBB models lends itself to high‐throughput screening of NPs and facilitates rapid optimization of experimental conditions.^[^ ^59^ ^]^ Coculture of astrocytes in the lower compartment can improve the tightness of in vitro BBB models.^[^ ^60^ ^]^ ECs–astrocyte–pericyte coculture model has also been developed as a triple‐coculture system. This arrangement contains ECs seeded on the support's upper surface, pericytes on the lower body, and astrocytes on the bottom of the culture wells (Figure 5D). A triple coculture model is a more reliable in vitro BBB model due to the higher transendothelial electrical resistance and the lower permeability.^[^ ^61^ ^]^

However, the transwell BBB models are static and do not offer the shear stress typically generated in vivo by the intravascular blood flow. Shear stress plays a substantial role in promoting and maintaining EC differentiation into a BBB phenotype.^[^ ^62^ ^]^ Researchers also developed dynamic BBB models with shear stress. The most representative dynamic BBB model would be microfluidic‐based models, which utilize the microfluidic technique to simulate blood flow and other dynamic interactions, offering a more physiologically relevant representation. They are cutting‐edge but require specialized equipment.^[^ ^63^ ^]^ Besides, brain spheroids or organoids represent advanced 3D cultures that closely mimic the architecture and cellular diversity of the BBB. They are typically derived from induced pluripotent or embryonic stem cells that self‐organize into structures resembling the brain microenvironment, including ECs, astrocytes, and neurons.​ These models offer several advantages, including the ability to study complex cell–cell interactions, disease‐specific modeling, and drug screening in a more physiologically relevant context. However, they are somewhat technically complex. Batch‐to‐batch variations and reproducibility in the BBB phenotype are the main considerations.^[^ ^64^ ^]^

In short, despite the development of various BBB models, the in vitro models currently used in research are far from ideal due to the complexity of the BBB. There is a need for improved models that balance the system's complexity and ease of development and use.^[^ ^58^ ^]^

### 2.3 Strategies for Crossing BBB

**Figure 6:**
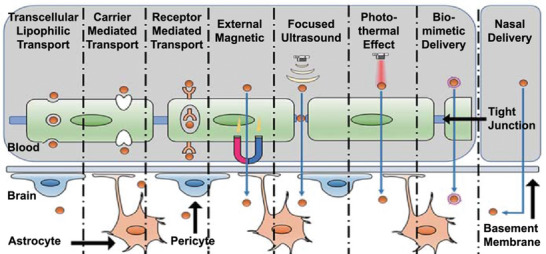
Schematic diagram of mechanisms for BBB crossing. Reproduced under the terms of the Creative Commons Attribution 4.0 International Public License.^[^ ^71^ ^]^ Copyright 2022, Guowang Cheng et al.

In recent years, the development of nanoplatforms designed to cross the BBB has emerged as a critical research area. These platforms employ various biological and physical mechanisms, as depicted in **Figure** **6**, to achieve drug delivery across the BBB.^[^ ^46^, ^65^ ^]^ Receptor‐mediated transport (RMT) is a common approach where nanoparticles are designed to mimic endogenous substances, such as lipoproteins, transferrin (Tf), and insulin, enabling them to be recognized by specific BBB receptors and transported into the brain.^[^ ^66^, ^67^ ^]^ Additionally, in terms of physical strategies, using magnetic nanoparticles guided by an external magnetic field has been shown to facilitate their passage through the BBB.^[^ ^68^, ^69^, ^70^ ^]^ Subsequent discussions will focus on different strategies to enhance the ability of nanoplatforms to cross the BBB.

#### 2.3.1 Transcellular Lipophilic Pathway (TLP)

TLP is a significant mechanism enabling lipophilic molecules to cross the BBB directly by diffusing through the cell membranes via passive diffusion, relying on the concentration gradient.^[^ ^72^ ^]^ A common strategy of TLP is to enhance the BBB permeation of small molecule drugs by chemically modifying them into a more lipophilic analog.^[^ ^73^, ^74^, ^75^ ^]^ However, in practice, very few small molecule drug candidates fit the dual criteria for lipid‐mediated free diffusion across the BBB, which are 1) MW < 400 Da threshold and 2) high lipid solubility. Nonetheless, one should ensure that structural modification on the lead compound does not affect the drug's stability or its degradation rate in the bloodstream.^[^ ^76^, ^77^ ^]^

An approach based on TLP involves lipophilic nanoparticles, such as liposomes and nanolipid carriers, designed to mimic the properties of biological membranes, enhancing their ability to permeate the BBB.^[^ ^78^ ^]^ However, this simple passive BBB transport mechanism was proved to be ineffective.^[^ ^79^ ^]^ Modifications are then proposed to enhance the transport of lipophilic nanoparticles across the BBB utilizing the active BBB transport mechanisms involving absorptive, carrier‐ or receptor‐mediated transcytosis.^[^ ^80^ ^]^ For example, taking advantage of the overexpression of transferrin (Tf) receptors in brain endothelial cells, Kong et al. demonstrated that Tf‐conjugated PEGylated liposomes delivered ostiole (Ost) to act against Aβ oligomer‐induced neurotoxicity. The in vivo study using transgenic (APP/PS1) mice found that the relative uptake efficiencies of Tf‐modified liposome was enhanced by four times, and the concentration efficiency was also enhanced by 3.38 times, respectively, compared to that of free Ost.^[^ ^80^ ^]^

#### 2.3.2 Carrier‐Mediated Transcytosis (CMT)

There are multiple surface transporters on the BBB that facilitate the transport of nutrients such as glucose and amino acids in a process called CMT.^[^ ^52^, ^81^ ^]^ Modifying drugs or nanomaterials with these transporters can enhance their ability to cross the BBB. A quantitative study of transporter expression levels in the human brain revealed that excitatory amino acid transporter (EAAT1) and glucose transporter 1 (GLUT1) are the most highly expressed solute transporters.^[^ ^82^, ^83^ ^]^ GLUT1 is far higher than other nutrient transport systems since the brain is in high demand of glucose as an energy source. Thus, GLUT1 is considered the most efficient transport system. Recently, Xie et al. modified glucose to polymeric nanomicelle (Glu‐PM‐Fab) for delivering bioactive antigen‐binding fragment antibodies (Fabs) against Aβ. Brain targeting was achieved by the interaction of glucose molecules on the nanomicelle surface with recycling GLUT1 proteins.^[^ ^84^ ^]^ They found that Glu‐PM‐Fab shows efficient and specific BBB penetration with a 42‐fold increase in delivery efficiency over free Fab.^[^ ^84^ ^]^

#### 2.3.3 Receptor‐Mediated Transcytosis

There are also various surface receptors, including Tf, insulin, leptin, lipoprotein, diphtheria toxin, folate, vasopressin, and glycation end products on the BBB, which specifically recognize large endogenous substances and transport them via RMT.^[^ ^66^ ^]^ RMT is the mostly used and most efficient mechanism for drug delivery.^[^ ^66^, ^85^, ^86^, ^87^, ^88^ ^]^ Most research in the past few decades has focused on ubiquitous targets known to be expressed on BBB cells, such as TfR, insulin receptors, members of the low‐density lipoprotein receptor (LDLR) family, and folate receptors. A large variety of antibodies and ligands capable of binding to such receptors have been developed. By conjugating nanomaterials with specific receptor antibodies or relevant ligands, their capacity to traverse the BBB can be enhanced.^[^ ^85^, ^86^, ^87^, ^88^ ^]^ TfR is the most widely studied and independently validated target protein for the RMT‐based brain delivery approach.^[^ ^66^, ^85^ ^]^ Recently, Jain et al. modified Tf on nanostructured lipid carriers (Tf‐NLCs) encapsulating two drugs, rivastigmine hydrogen tartrate and resveratrol, for AD treatment.^[^ ^89^ ^]^ Their study found that TF‐NLCs demonstrated a significantly slow release compared to NLCs and revealed significantly higher (≈1.7‐fold) brain uptake of Tf‐NLCs compared to NLCs in vivo after intraperitoneal administration.

#### 2.3.4 Adsorption‐Mediated Transport (AMT)

Since the luminal side of the BBB features a negative charge (due to proteoglycans), electrostatic interactions are triggered whenever a positively charged substance encounters the plasma membrane surface. AMT involves the endocytotic internalization of macromolecules via this pathway, followed by their subsequent passage through the BBB.^[^ ^90^ ^]^ Therapeutically, AMT can be achieved in one of two ways: i) by building cationic surface charge on NP, such as modifying NP with small molecules bearing positive charge like chitosan and 3‐aminopropyltriethoxysilane, or ii) by conjugating NP with a positively charged moiety, such as a cell‐penetrating peptide (CPP).^[^ ^91^ ^]^ CPPs are short targeting vectors that typically consist of less than 30 amino acids, and CPPs cross the membrane relying on positively charged amino acids interacting with the negatively charged membrane.^[^ ^92^ ^]^ Among all CPPs, the HIV‐1 tat (TAT) protein is the most studied cell membrane peptide used for brain targeting, which is derived from 48 to 60 amino acids in the Tat column of the HIV.^[^ ^93^ ^]^ Earlier studies showed that the smallest domain of TAT protein playing a membrane penetrating role is the amino acid sequence TAT at positions 49–57, composed of 9 amino acids and containing six arginine residues.^[^ ^94^ ^]^ The guanidine group of arginine interacts with the negatively charged carboxylic acid and phosphate groups on the cell membrane to form hydrogen bonds, thus crossing the cell membrane and entering the cell. Recently, Feng et al. constructed a TAT‐modified chondroitin sulfate (CS) gold nanoparticle (TAT‐CS@Au) delivery system to enhance CS delivery to the brain for AD treatment.^[^ ^95^ ^]^ In SH‐SY5Y cells, Cy5‐TAT‐CS@Au shows significantly enhanced cell uptake compared with free Cy5‐PEG‐SH, which stemmed from TAT‐mediated cellular endocytosis and the transmembrane transport ability of gold nanoparticles (AuNPs).

Limitations of AMT include its lack of selectivity for the nonspecific adsorption in the blood vessels of other organs and its potential to increase BBB vascular permeability due to the possibly toxic effects of positively charged compounds, such as CPPs, when administered in large amounts.^[^ ^91^, ^96^ ^]^

#### 2.3.5 Nasal Delivery

Other alternative delivery routes, like intranasal delivery of nanomedicines, have also gained much attraction because they are noninvasion as they bypass the BBB. Drugs could be directly delivered to the brain via the olfactory region, thereby enhancing the drug's bioavailability and activity.^[^ ^97^ ^]^ The use of NPs can enhance this effect since it can potentially enable higher payload capacities, selective targeting capability, controlled release, and increased drug retention in the nasal mucosa.^[^ ^98^ ^]^ Xia et al. found that surface‐modified NPs yield a greater brain drug concentration when delivered by the intranasal route than by conventional oral and intravenous routes.^[^ ^99^ ^]^ In another study, Viola et al. designed Aβ oligomer‐specific antibodies conjugated Fe_3_O_4_ magnetic nanoparticles as the MRI contrast platform for diagnosis of AD through intranasal administration,^[^ ^100^ ^]^ confirming that particles can be delivered by intranasal inoculation and specifically bind to the target in the brain.^[^ ^100^ ^]^ In another work, Zhang et al. used solanum tuberosu lectin‐functionalized polyethylene glycol–polylactide‐polyglycolide (PEG–PLGA) NPs for effective delivery of basic fibroblast growth factor (bFGF) to the brain by intranasal injection,^[^ ^101^ ^]^ which could effectively facilitate direct transport of bFGF into the rat brain with reduced peripheral adverse effects via intranasal administration.

Although nasal delivery offers advantages, it also has limitations such as inconsistent clinical results, anatomical and physiological barriers, and rapid clearance from the cerebrospinal fluid.^[^ ^102^ ^]^ These challenges highlight the need for further research and innovation to establish the efficacy and safety of intranasal drug delivery for clinical use.

#### 2.3.6 Membrane Coating

As an alternative strategy, camouflaged drug delivery systems utilizing biomimetic materials have been developed to facilitate the traversal of the BBB for targeted drug delivery to the brain.^[^ ^103^, ^104^, ^105^, ^106^, ^107^, ^108^, ^109^, ^110^ ^]^ The concept of biomimetic drug delivery was initially proposed by Zhang et al. in 2011, who demonstrated that nanoparticles coated with red blood cell membranes exhibited significantly increased in vivo retention times. Since then, an increasing number of biomimetic nanosystems for drug delivery have been developed, including membrane‐enabled technology,^[^ ^111^, ^112^, ^113^ ^]^ extracellular vesicles bionic methods,^[^ ^45^, ^114^, ^115^ ^]^ virus‐inspired synthesis, and bacteria bionic strategies.^[^ ^116^ ^]^ Among these, using cell membrane‐modified nanoparticles has become the most extensively explored approach for drug delivery to the brain.^[^ ^44^ ^]^ Researchers have devised nanoparticulate systems with excellent characteristics. For example, NPs coated with red blood cell membranes have been prepared to enhance blood circulation.^[^ ^110^, ^117^ ^]^ Other drug delivery systems utilize coatings of various cell membranes like brain tumor cells and immune cells, modified with specific antibodies or ligands that can target the brain.^[^ ^112^, ^113^ ^]^

Similar to cell membranes derived from different kinds of cells, extracellular vehicles (EVs) are nanometer‐sized membrane‐surrounded vesicles secreted by most cells and contain lipids, proteins, and various nucleic acid species of the source cell, which act as major conduits of long‐distance communication to carry bioactive molecules and deliver them to recipient cells.^[^ ^118^ ^]^ EVs are generally classified into three distinct populations based on their biogenesis: exosomes, microvesicles, and apoptotic antibodies.^[^ ^119^ ^]^ The ability to transport biomolecules to recipient cells has made them attractive for drug delivery purposes. EVs have an excellent drug‐carrying capacity and can accommodate hydrophobic and hydrophilic drugs. The biological origin of EVs will overcome the drawbacks related to synthetic NPs. The benefits of extended blood circulation half‐life, excellent BBB traversal, lower toxicity, hypo‐immunogenicity, and reflection of the “inheritance” from the parent cell and cellular affinity make exosomes an excellent drug delivery vehicle for various diseases.^[^ ^118^ ^]^ For example, the antioxidant curcumin has been loaded by incubation in macrophages derived EVs to improve the solubility and bioavailability of the drug for AD treatment and increase drug penetration across the BBB by the lymphocyte function‐associated antigen one and endothelial intercellular adhesion molecule 1 inherited from the parent cell.^[^ ^120^ ^]^

#### 2.3.7 External Physical Stimuli‐Mediated Strategies

In addition to biological avenues, external physical stimuli such as light, ultrasound, and magnetic fields also hold promise for enhancing drug delivery across the BBB. For example, magnetic nanoparticles can be directed using external magnetic fields to release their drug payload near the BBB or even to induce localized BBB disruptions.^[^ ^68^ ^]^ Tan and co‐workers wrapped magnetic NPs in PEG and polyethyleneimine (PEI) and intravenously injected them into mouse models, followed by providing an external magnetic field to the brain. The authors showed that the iron content in the brain of the mice was significantly higher in the magnetic field treatment group than in the control group without a magnetic field, indicating that the external magnetic field successfully facilitated the accumulation of particles in the brain.

Ultrasound is a technique that can noninvasively focus deep into the body using an ultrasound field.^[^ ^121^ ^]^ Since the 1940s, ultrasound has been noted for noninvasive ablation in the brain.^[^ ^122^, ^123^ ^]^ Previous studies showed that focused ultrasound can stimulate local, reversible opening of the BBB when used in conjunction with microbubble contrast agents.^[^ ^124^ ^]^ Qin and co‐workers constructed a microbubble delivery system Qc@SNPs‐MB by embedding quercetin‐modified sulfur NPs (Qc@SNPs) in microbubbles MB.^[^ ^124^ ^]^ Qc@SNPs‐MB was destroyed instantly when exposed to ultrasonic pulses, and it enhanced the permeability of the blood vessels, resulting in the brief opening of the BBB owing to the “sonoporation” effect. Simultaneously, Qc@SNPs were released from the outer shell of the microbubbles and entered the brain across the open BBB, accumulating in the brain parenchyma.^[^ ^124^ ^]^

The photothermal technique is another strategy that can transiently open the BBB. Xing and co‐workers designed NIR light‐responsive NPs conjugated with photothermal polymer PDPP to inhibit and disaggregate Aβ_42_ fibrillation.^[^ ^125^ ^]^ Interestingly, upon NIR light irradiation, benefiting from the high photothermal conversion efficiency of PDPP, NPs generate local heat and effectively promote BBB permeability. Additionally, several studies use ruthenium nanomaterial, Nb2C MXenzyme, and Fe_3_O_4_ nanoparticles, highlighting their excellent photothermal conversion capabilities to ameliorate and enhance the BBB permeability for AD treatment effectively.^[^ ^125^ ^]^

Opening the BBB might allow effective delivery of therapeutic agents to the brain, but it also risks introducing unwanted substances like pathogens and neuroinflammatory agents. Using External physical stimuli‐mediated strategies is not yet a mainstream approach.

## 3 Nanomaterials‐Based Modification for BBB Crossing

Nanomaterials have been widely developed to transport therapeutic drugs through the BBB due to their apparent advantages, such as relatively high drug loading content, enhanced stability, prolonged blood circulation time, controlled drug release, and targeting effect. The size, zeta potential, and hydrophilicity of nanomaterials play important roles in their fate in vivo.^[^ ^126^ ^]^ Ideal properties for nanoparticle‐based brain drug delivery typically include: i) being nontoxic, biodegradable, and biocompatible, ii) preferred size ranges from 20 to 200 nm, iii) physically stable and affording prolonged blood circulation time in the blood, iv) BBB crossing and brain targeted delivery with controlled drug release capability.^[^ ^127^, ^128^ ^]^ We have summarized the NPs mentioned in the review in **Table** **1**.

**Table 1:** Summary of NPs with enhanced BBB penetration for AD treatment and diagnosis.

Types	Materials	Size [nm]	BBB crossing strategies	Targeting agents	Active drugs	Diagnostic/therapeutic pathways	Refs.
Lipid‐based	Liposomes	110–170	AMT	WGA	NGF/Cur	Protect neurons, alleviate oxidative stress and inflammation	[148]
		110	CMT	GSH	VHH‐pa2H	Target Aβ	[151]
		196.3 ± 7.09	RMT	Tf	α‐Mangostin	Protect neurons, alleviate oxidative stress and inflammation	[152]
		125–135	RMT	Tf/PA	Pep63/PA	Inhibit Aβ aggregation, enhance Aβ clearance	[153]
		110	RMT	Lf	NGF	Protect neurons	[155]
		105–110	RMT/AMT	Lf/RMP‐7	Quercetin	Inhibit Tau phosphorylation	[156]
		173.6 ± 1.16	CMT/AMT	MAN/TAT	miRNA‐195	Inhibit Aβ aggregation and Tau phosphorylation, suppress microglia activation	[157]
	SLNs	<200	RMT	PS80	Quercetin	Alleviate oxidative stress	[184]
		168–189	RMT	OX26 mAb	Resveratrol	Inhibit Aβ aggregation	[192]
		147.5 ± 0.76	RMT	ApoE	Donepezil	Improve neurotransmitter deficiency (AChEI)	[193]
	NLCs	186	TLP	None	Berberine	Improve neurotransmitter deficiency, reduce Aβ levels, enhance antioxidative activity	[201]
		103.8 ± 0.6	RMT	Lf/LDL‐mimic NLC	Cur	Alleviate oxidative stress	[202]
		90.5 ± 0.2	RMT	PS80	Cur	Alleviate oxidative stress	[203]
		90.5 ± 0.2	RMT	Tf	Rapamycin	Decrease Aβ formation; reduce oxidative stress and neuroinflammation	[204]
Polymer‐based	PNPs	250 ± 30	RMT	PS80	NGF	Improve neurotransmitter deficiency	[227]
		153.2 ± 13.7	Nasal delivery	Lf	Huperzine A	Improve neurotransmitter deficiency (AChEI)	[228]
	Nanogels	9 ± 24	RMT	Insulin	Insulin	Inhibit intrinsic apoptotic pathway; reduce oxidative stress	[236]
		122	RMT	Angiopep‐2	Oxytocin	Inhibit microglial activation and reduce inflammatory cytokine levels	[238]
	Micelles	53	RMT	Lf	CLA	Reduce oxidative stress, inflammation, apoptosis, and AChE activity, inhibit Aβ deposition	[242]
		65	AMT	Ab peptide	PEG‐LysB/Cur	Scavenge ROS, inhibit Aβ aggregation	[243]
		41.05 ± 1.20	AMT	TPL	Rapamycin	Enhance clearance of Aβ and p‐Tau	[244]
	Dendrimers	131.72 ± 4.73	RMT	Lf	Memantine	Improve neurotransmitter deficiency	[249]
		6 ± 0.1	CMT	Histidine‐maltose	Histidine‐maltose	Inhibit Aβ fibril formation	[250]
		21	AMT	Ab peptide	ROS‐responsive dendrimer/p‐Nrf2	Eliminate ROS, alleviate microglia activation	[251]
	Polymeric nanocomposites	None	Nasal delivery	None	MB/BP NSs	Suppress Tau neuropathology; restore mitochondrial function; alleviate neuroinflammation	[253]
		153.4	RMT	Angiopep‐2	AIE molecules	Inhibit the Aβ fibrils formation; degrade Aβ fibrils; relieve the ROS and inflammation	[254]
		31.02	Nasal delivery		HDL‐Disc	Enhance both central Aβ clearance and peripheral Aβ clearance	[255]
Metal‐based	AuNP	3.3	TLP	None	GSH/AuNP	Inhibit Aβ aggregation	[264]
		5	TLP	None	Bucladesine	Improve acquisition and retention of spatial learning and memory of AD mice	[265]
		5	TLP	None	Anthocyanins	Inhibit Aβ plaques	[266]
		80	RMT	Tf	None	Probe RMT mechanism	[274]
		12	RMT	THR peptide	None	Prove RMT can enhance BBB crossing	[276]
		4	Nasal delivery	WGA‐HRP	None	Reduce the number of drugs and extend the effective time of drugs	[277]
	AuNS	105		Pen peptide	Ru (II)	PDT	[279]
	ZnO NP	47	TLP	None	AChEI	Reduce hydrolysis of neurotransmitter ACh and improve cognitive function	[280]
	ZnSe	150	RMT	Ang peptide	Exchange with endogenous Cu^2+^	Inhibit ROS generation and protect neuronal cells from necrosis and apoptosis. PTT	[282]
	SeNP	96.11 ± 3.45	RMT	LPFFD and TGN	Cur	Suppress extracellular Aβ fibrillation	[287]
		55	Opening of BBB	None	Bor	Facilitate SeNP accumulation in the brain	[288]
		95	RMT	Peptide‐B6	Sialic acid	Inhibit and disaggregate Aβ fibrils	[289]
		84.19 ± 1.76	RMT	Tg peptide	Dihydromyricetin	Diminish Aβ aggregation	[290]
		100 ± 30	TLP	Arginine‐dehydrophenylalanine	None	Disaggregate Aβ fibrils	[291]
	RuNP	140	TLP	None	NGF	Repair neuronal damage and inhibit p‐Tau‐related pathogenesis	[297]
	RuO_2_	50	Opening of BBB	None	Bor	Reduce oxidative stress, inhibit Aβ aggregation, decrease inflammation	[298]
	CeO_2_ NP	20–40	TLP	None	CeO_2_	Mitigate active gliosis and repair mitochondrial ROS‐induced damage	[299]
		160	TLP	None	EuCeO_2_	Attenuate LPS‐elicited proinflammatory microglial responses	[300]
	SPIO NP	30–50	TLP	None	Quercetin	Enhance BBB crossing capability and recover memory impairment	[307]
		45–50	TLP	None	Carbazole‐based oligomer‐selective cyanine	Slow signal decline and enhance Aβ signal in MRI	[308]
		180	RMT	CRT peptide	Cur	Induce neuroprotection and neurogenesis	[309]
		3	RMT	T7 peptide	MAM	Enhance MRI signal	[310]
Silica NPs	SiO_2_ NP	<100	AMT	None	None	Prove PEG–PEI coating can greatly enhance uptake	[317]
		20, 50, 100	RMT	Lf	None	Boost uptake	[320]
		50	RMT	Ri7 peptide	None	Augment uptake	[321]
		65.2± 4.9	TLP	None	Cyclen	Inhibit metal‐induced Aβ toxicity	[322]
		100	RMT	Tf	HI‐6	Inhibit AChE	[323]
		80–100	CMT	Lipid coating	BBR	Inhibit Aβ aggregation	[324]
		100	RMT	PS‐80	RV	Slow drug release and enhance BBB penetration	[325]
		100	TLP	None	Chiral helicases	Inhibit formation of β‐sheet structures and fibrils	[326]
CNs	Carbon nanotubes		RMT	Gadolinium L2	None	Enhance uptake	[328]
	Mesoporous carbon nanoparticle	89 ± 2	RMT	RVG peptide	PX	Reduce p‐Tau and inhibit Aβ aggregation	[331]
Membrane coating	RBCM‐coated lipid carrier	<160	RMT	TPP	Resveratrol	Mitigate ROS, improve memory impairment in mice	[104]
	RBCM‐coated human serum albumin NPs	120	NA	T807/TPP	CUR	Mitigate ROS, suppress neuronal death	[333]
	RBCM‐coated CQD	169.9±2.9	PTT	None	PDA	Inhibit Aβ aggregation, disintegrate Aβ fibrils, scavenge ROS, and mitigate neuroinflammation	[334]
	RBCM‐coated polycaprolactone nanoparticles	<240	RMT	TGN peptide	Curcumin	Reverse cognitive function, protect neuro, and mitigate neuroinflammation	[335]
	Neutrophil‐like cell‐membrane‐coated CuxO	≈100	NA	Aβ‐targeting pentapeptide KLVFF	None	Clear peripheral Aβ	[342]
	MM‐coated SLNs	<140	RMT	RVG29/TPP	Genistein	Decrease Aβ deposition, alleviate ROS, prevent abnormal glial activation and neuroinflammation	[343]
	MM‐coated liposome	111.27 ± 9.64	Bypass the BBB	None	Oxytocin	Inhibit neuronal apoptosis, enhance synaptic plasticity	[106]
	MM‐coated MoS_2_ QDs	125.62 ± 4.1	PTT	None	None	Eliminate ROS and resist Aβ deposition	[336]
	Neural stem cell membrane‐coated AgAuSe QDs	44	RMT	RVG	Bexarotene	Realize in vivo imaging of brain, reduce soluble Aβ	[108]
	Exosomes‐encapsulated Pt NPs	118	RMT/PTT	RVG	Resveratrol	Scavenge ROS, protect mitochondria, attenuate glial cell activation, reduce Aβ plaques	[337]
	Exosome	117.4 ± 10.5	NA	LFA‐1, ICAM‐1	Curcumin	Inhibit Tau phosphorylation, enhance neuronal rescue	[338]
MoF‐based	PCN‐224 NPs	70	NA	None	None	Reduce Aβ‐induced toxicity in cells	[358]
	UiO‐66(Zr) NPs	500.80 ± 16.63		NA	Magnolol	Enhance neuroprotective activities	[345]
	CeNPs‐loaded MIL‐100(Fe)	None	NA	None	siSOX9/Retinoic acid	Ameliorate cognitive impairment in mice	[339]
	CeONP‐loaded ZIF‐8 NPs	106	NA	AβO aptamer	Resveratrol	Visualize intracellular AβO, degrade Aβ aggregates, reduce AβO‐induced ROS, and protect neurons from apoptosis in cells	[43]

Abbreviation: Red blood cell membrane (RBCM), macrophage membrane (MM), rabies virus glycoprotein (RVG), lymphocyte function‐associated antigen 1 (LFA‐1), and endothelial intercellular adhesion molecule 1 (ICAM‐1), peptide TGNYKALHPHN (TGN).

### 3.1 Lipid‐Based

First identified in 1961 by Müller and Gasco, lipid NPs have been praised for their biocompatibility, biodegradability, proficiency in encapsulating lipophilic drugs, and facile alteration with targeting ligands.^[^ ^129^, ^130^ ^]^ Their therapeutic potential was swiftly acknowledged postdiscovery; however, it is only in recent decades that they have been critically appraised as a promising vehicle for pharmaceuticals intended for the CNS, especially AD.^[^ ^131^, ^132^ ^]^ Some research findings showed that small lipophilic molecules of less than 400 Da can freely diffuse across the BBB endothelium.^[^ ^133^ ^]^ Due to their size and inherent lipophilic properties, lipid NPs, therefore, can interact as a drug carrier molecule with the BBB and its components and cross the BBB via different mechanisms.^[^ ^130^, ^134^, ^135^, ^136^ ^]^ Presently, three types of lipid‐based nanomaterials have been engineered for drug delivery purposes, specifically, liposomes, solid lipid NPs (SLNs), and NLCs.^[^ ^137^ ^]^

#### 3.1.1 Liposomes

**Figure 7:**
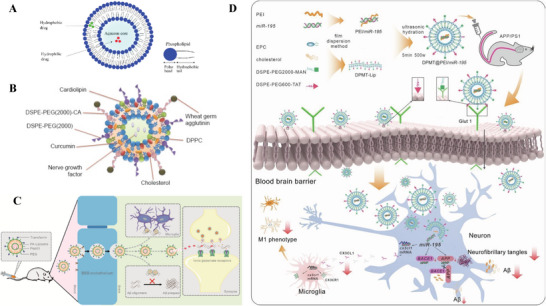
A) Scheme of a simple liposome with an aqueous core and phospholipid bilayer. Reproduced under the terms of the Creative Commons Attribution 4.0 International Public License.^[^ ^97^ ^]^ Copyright 2022, Ayub et al. B) Illustration of the structure of the WGA–CL–NGF–CUR–liposome. Reproduced under the terms of the Creative Commons Attribution 4.0 International Public License.^[^ ^148^ ^]^ Copyright 2015, Kuo et al. C) Diagram of Tf‐Pep63‐Lip liposomes for combined AD therapy. Reproduced under the terms of the Creative Commons Attribution 4.0 International Public License.^[^ ^153^ ^]^ Copyright 2021, Yang et al. D) Illustrative overview of DPMT@PEI/miR‐195 synthesis and its mechanism against AD pathology. Reproduced with permission.^[^ ^157^ ^]^ Copyright 2024, Elsevier.

Liposomes ranging from 50 to 500 nm are spherical vesicles comprising one or more lipid bilayers resulting from emulsifying natural or synthetic lipids in an aqueous medium, as depicted in **Figure** **7**A.^[^ ^97^ ^]^ Being amphiphilic entities, liposomes possess a hydrophilic core with a lipophilic tail,^[^ ^97^ ^]^ which endows them with the capability to encapsulate both hydrophilic and hydrophobic drugs alongside bioimaging agents to enhance therapeutic and diagnostic strategies, offering a promising solution to bypass the BBB's restrictive nature. Their modifiability for targeted delivery enhances their suitability, allowing for developing strategies that increase their circulation time and promote specific uptake by brain tissues. Therapeutically, liposomes can bolster drugs’ biocompatibility, stability, and controlled release while enabling enhanced permeability and targeted delivery by modification.^[^ ^138^, ^139^, ^140^ ^]^ The PEGylation of liposomes further enhances the efficacy of encapsulated molecules by reducing the opsonization and reticuloendothelial system (RES) clearance in vivo. This not only prolongs drug retention by extending blood circulation but also facilitates therapeutic accumulation at the requisite site, thereby mitigating side effects.^[^ ^141^ ^]^ In relation to AD therapy, liposomes can be tailored to target individual or multiple pathological targets via the modification with brain‐penetrating peptides in conjugation with Aβ‐targeting ligands, such as phosphatidic acid (PA), curcumin (Cur), and a retro‐inverted peptide that inhibits Aβ aggregation.^[^ ^79^ ^]^ It has been suggested that Aβ preferentially inserts into anionic phospholipids incorporated into liposomes, which could potentially exert a protective effect by facilitating the removal of toxic Aβ.^[^ ^142^, ^143^ ^]^ Currently, liposomes represent one of the most extensively studied NP types for cerebral delivery, suggesting their great potential for clinical applications.^[^ ^131^ ^]^ The integration of several modifications into multifunctional liposomes represents an area of ongoing research, highlighting the unceasing interest in this field.^[^ ^79^ ^]^

Possible mechanisms for the transport of liposomes across the BBB have been highly debated.^[^ ^144^ ^]^ An initial theory was that the phospholipid bilayer of the liposomes on its own might facilitate transportation across various biological membranes, including the BBB, but this simple mechanism proved to be ineffective.^[^ ^79^ ^]^ Modifications proposed for enhancing the transport of liposome carriers across the BBB utilize the existing active transport mechanisms involving absorptive, carrier‐ or receptor‐mediated transcytosis.^[^ ^145^ ^]^ Since absorptive methods take advantage of the BBBs’ negative charge, a variety of proteins or peptides with a positive charge in the physiological environment can be transported across the BBB via AMT.^[^ ^79^, ^146^ ^]^ Wheat germ agglutinin (WGA) is a basic peptide with distinct carbohydrate specificity and is efficient in the delivery of drugs across the BBB.^[^ ^147^ ^]^ In 2015, Kuo and Lin developed a WGA‐conjugated liposome with cardiolipin (CL) incorporated to permeate the BBB, as shown in Figure 7B. The WGA‐conjugated and CL‐incorporated liposomes (WGA‐CL‐liposomes) were used to transport nerve growth factor (NGF) and Cur across a monolayer of HBMECs, which are regulated by human astrocytes. The results in vitro showed that the NGF/Cur@CL‐liposomes‐WGA protect SK‐N‐MC cells against apoptosis induced by Aβ_42_ fibrils.^[^ ^148^ ^]^ Helm and Fricker studied the transportation of cationized bovine serum albumin (cBSA)‐functionalized liposomes in vivo with rat models and in vitro with porcine brain capillary ECs (pBCEC). They showed that the conjugation of the liposome with cBSA was the key to the uptake by the pBCEC, compared to the fact that no adsorption was observed for the liposome alone or conjugated with native BSA. However, the nonspecific uptake of the cationic liposomes by peripheral tissues and their binding to serum proteins has meant that a toxic dose would often be required to reach therapeutic efficacy, thus limiting therapeutic potential.^[^ ^149^ ^]^ Glutathione (GSH), actively transported across the BBB via a sodium‐dependent GSH transporter highly expressed on the BBB epithelium, can be utilized to facilitate liposome passage through the BBB by anchoring it onto the liposome surface.^[^ ^150^ ^]^ “G‐Technology” is a strategy involving PEGylated liposomes with glutathione (GSH‐PEG)‐mediated delivery across the BBB into the brain, and this has shown some success in an Aβ‐targeting antibody fragment delivery in model mice of AD, but receptor saturation limits this system's effectiveness.^[^ ^151^ ^]^ Ultimately, RMT has great potential for success since there is the possibility of targeting one or more of the many different receptors at the BBB by using their respective ligands. Chen et al. prepared a Tf‐modified liposome to deliver α‐Mangostin, a neuroprotector against Aβ oligomers‐induced toxicity, for AD treatment, which showed a fourfold higher cellular uptake in the brain.^[^ ^152^ ^]^ Yang et al. developed a Tf‐modified liposome to deliver neuroprotective Pep63, a 10‐mer peptide that exerts a neuroprotective effect on synaptic plasticity and memory, and PA, which were incorporated to enhance the Aβ‐binding ability for AD treatment. They discovered that administration of Tf‐Pep63‐Lip could significantly reduce the Aβ burden and improve cognitive deficits in AD mouse model with the combined effects of PA and Pep63 in the following ways (Figure 7C): 1) inhibiting Aβ_42_ aggregation; 2) disaggregating Aβ fibrils; 3) facilitating Aβ chemotaxis/phagocytosis of microglia; and 4) rescuing NMDA receptors trafficking.^[^ ^153^ ^]^ Lf‐functionalized liposomes may have the potential to be more efficient, as the expression of the LfR on microvessels and neurons is increased in AD,^[^ ^154^ ^]^ allowing more effective targeting. In 2014, Kuo and Wang developed Lf‐modified liposomes for β‐neuron growth factor delivery in neuron‐like SK‐N‐MC cells. The results showed that the liposomes modified with Lf could cross the BBB and inhibit Aβ‐induced neurotoxicity.^[^ ^155^ ^]^ Then, they propelled the Lf‐modification method forward in 2017 by establishing a multifunctional liposome Qu@liposomes‐RMP‐7‐Lf to permeate the BBB model of HBMECs.^[^ ^156^ ^]^ Recently, liposomes with dual modifications to facilitate BBB crossing have been designed. Most recently, Su et al. formulated liposomes encapsulating PEI/miR‐195 complex (DPMT@PEI/miR‐195) that was engineered through dual modifications to contain P‐aminophenyl‐alpha‐d‐mannopyranoside (MAN) and cationic cell‐penetrating peptide TAT. The MAN molecules allow liposomes to attach to the Glut1 on ECs, facilitating initial interaction via CMT. TAT peptides further enhanced the liposomes crossing BBB via AMT. These MAN and TAT dually modified liposomes cross BBB with higher efficiency. MicroRNA‐195, the first miRNA‐based drug for AD therapy, targets Aβ, Tau, and microglia polarization simultaneously, alleviating the cognitive decline in mice models (Figure 7D).^[^ ^157^ ^]^

Liposomal encapsulation of compounds improves BBB penetration via intranasal delivery, protecting them from degradation and facilitating their transport across the mucosal barrier.^[^ ^79^ ^]^ Zheng et al. have found that a “β‐sheet” breaker peptide, H102, encapsulated in liposome, can be effectively released in the brain via intranasal administration, maintaining an area under the plasma concentration time curve (AUC) almost three times greater than that obtained by a peptide solution.^[^ ^158^ ^]^

The liposomes are often regarded as the first generation of drug delivery systems, with their first report dating back as far as 1971, and the liposome Doxil loaded with doxorubicin (Dox) modified by polyethylene glycol was approved by the U.S. FDA as the first nanomedicine for clinical application in 1995.^[^ ^159^ ^]^ Despite numerous liposomal formulations under clinical trials in the USA for various conditions, their use for brain‐specific drug delivery remains limited, and their clinical application is still under the preclinical stage.^[^ ^160^, ^161^ ^]^ Some of the liposomal drugs for brain drug delivery are listed in **Table** **2**.^[^ ^162^ ^]^

**Table 2:** Status of liposomal formulations in clinical trials for brain drug delivery.^[^ ^162^ ^]^

Compounds (trade name)	Therapeutic use	Status	Refs.
Doxorubicin (Doxil)	Glioblastoma multiformae	Phase ll	Beier et al., 2009; Ananda et al., 2011
	Pediatric brain tumor	Phase I	Beier et al., 2009; Ananda et al., 2011
Daunorubicin (Daunoxome)	Pediatric brain tumor	Phase I	Lippens, 1999
Doxorubicin (Myocet)	Glioblastoma multiformae	Phase ll	Ananda et al., 2011
Cytarabine (Depocyt)	Lymphomatus meningitis	FDA approved	Benesch and Urban, 2008
Amphotericin B (AmBisome)	Cryptococcal meningitis	FDA approved	Loyse et al., 2013
Amphotericin B (Abelcetâ)	Cryptococcal meningitis	FDA approved	Loyse et al., 2013

Although liposomes hold considerable promise in AD therapy, their use in drug delivery systems is not perfect. Challenges include instability, attributed to the propensity of liposomes to accumulate, resulting in premature drug leakage and rapid clearance by the mononuclear phagocyte system. Additionally, the production of liposomal drug‐delivery systems entails substantial costs, particularly those with specialized modifications for targeted delivery, imposing significant constraints on their broader applications. Furthermore, the long‐term storage and limited drug‐loading capacity of liposomes can also be a concern.^[^ ^97^, ^163^, ^164^ ^]^

#### 3.1.2 Solid Lipid Nanoparticles

**Figure 8:**
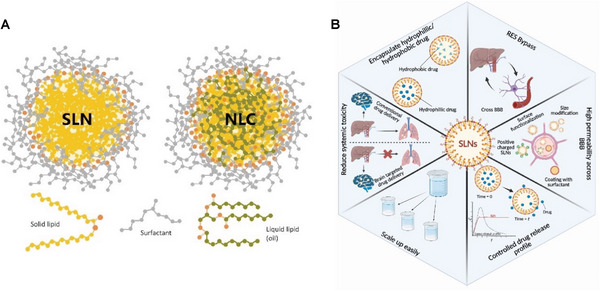
A) Scheme of an SLN and an NLC sterically stabilized with a neutral surfactant (gray). Reproduced under the terms of the Creative Commons Attribution 4.0 International Public License.^[^ ^181^ ^]^ Copyright 2020, Montoto et al. B) Advantages of employing SLNs in AD drug delivery and treatment. Reproduced under the terms of the Creative Commons Attribution 4.0 International Public License.^[^ ^195^ ^]^ Copyright 2023, Naser et al.

SLNs constitute a colloidal carrier system characterized by a solid lipid core of high melting point, enveloped by an aqueous surfactant to enhance stability, as depicted in **Figure** **8**A.^[^ ^165^ ^]^ Typically, SLNs encompass 0.1% to 30% (w/w %) solid lipids dispersed in an aqueous medium, stabilized by a surfactant in concentrations oscillating from 0.5% to 5% (10–1000 nm diameter size range).^[^ ^166^, ^167^ ^]^ This solid lipid core exhibits the capacity to incorporate both hydrophobic and hydrophilic drugs.^[^ ^168^, ^169^ ^]^ However, recent research indicates that the loaded drug may predominantly adhere to the surface of the carrier matrix rather than being integrally embedded within the solid core.^[^ ^170^, ^171^ ^]^ Facilitating their transport in body fluids, these nanocarriers present a various size range, which depends on the method of preparation and the specific formulation used.^[^ ^172^ ^]^ Introduced in 1991,^[^ ^173^ ^]^ SLNs are considered as a superior alternative to conventional colloid systems such as emulsions, liposomes, and polymeric NPs.^[^ ^174^ ^]^ The employment of solid lipids, as opposed to liquid lipids, bolsters the stability of incorporated labile pharmaceuticals, safeguarding them from degradation.^[^ ^175^ ^]^ Moreover, it facilitates a more regulated release kinetics owing to the slow digestion of the dense lipid matrix.^[^ ^174^, ^176^ ^]^ Given that SLNs are constructed from a physiological solid lipid emulsion system, organic solvents are largely avoided, leading to superior biocompatibility and diminished systemic toxicity compared to polymeric nanoparticles.^[^ ^177^ ^]^ Economically, SLNs are favorable due to their low raw material and production costs. Their excellent physicochemical stability and suitability for cost‐effective commercial sterilization and lyophilization further underscore their advantages.^[^ ^178^ ^]^ The advantages of employing SLNs in AD drug delivery and treatment can be summarized as shown in Figure 8B.

SLNs have lately received new consideration as a possible drug delivery system for brain targeting because they are one of the safest and least expensive drug carriers.^[^ ^179^, ^180^, ^181^ ^]^ They have been employed to encapsulate existing pharmaceuticals, thereby enhancing bioavailability and therapeutic efficacy in AD treatment.^[^ ^182^, ^183^ ^]^ This is achieved by optimizing their size to <200 nm and modifying their surface with hydrophilic polymers or surfactants such as PEG, Brij 78, Poloxamer F68, and Polysorbate (20, 60, 80) to extend circulation time and bypass RES recognition, respectively.^[^ ^179^ ^]^ For example, antioxidants such as quercetin,^[^ ^184^ ^]^ piperine,^[^ ^185^ ^]^ sesamol,^[^ ^186^ ^]^ and curcumin,^[^ ^187^ ^]^ FDA‐approved drugs like galantamine,^[^ ^188^ ^]^ and other therapeutic compounds like Huperzine A,^[^ ^189^ ^]^ nicotinamide,^[^ ^190^ ^]^ and erythropoietin^[^ ^191^ ^]^ have demonstrated superior bioavailability and therapeutic efficacy in in vivo studies compared to the standalone drug when loaded into SLNs.

Ligands have been utilized to modify SLNs, enhancing their selectivity and cerebral targeting capability via specific binding to surface receptors expressed on BBB cells. Loureiro et al. developed an antitransferrin receptor monoclonal antibody (OX26 mAb) modified SLNs encapsulating resveratrol from grape skin and seed extracts for AD treatment.^[^ ^192^ ^]^ The monoclonal antibody (mAb) type OX26 specifically binds to the TfR expressed on BBB cells, thus facilitating BBB crossing via RMT. Resveratrol was shown to inhibit Aβ aggregation here, having the potential to treat AD. Experiments on human brain‐like ECs showed that the cellular uptake of the OX26 SLNs was substantially more efficient than that of control SLNs and SLNs functionalized with an unspecific antibody. In 2021, Apolipoprotein E (ApoE)‐targeting SLNs were engineered for the delivery of Donepezil (DON), an anti‐Alzheimer's drug. ApoE associates with lipids to form lipoproteins which have high affinity to the LDLRs on both brain ECs and neurons, play a key role in the transport and uptake of cholesterol in the brain by RMT. In vitro study findings indicated that the DON@ApoE‐SLNs enhanced drug delivery with a favorable release profile in both cultured human brain ECs and neurons, as well as elevated the permeability across the BBB model.^[^ ^193^ ^]^

Still, despite their inherent advantages, SLNs are subject to several limitations, including a limited capacity for component integration, potential degradation, or leakage of components during storage due to crystallinity fluctuations of solid lipids, suboptimal loading capacity for certain active compounds, and a significant water requirement for dissolution.^[^ ^194^ ^]^

#### 3.1.3 Nanostructured Lipid Carriers

**Figure 9:**
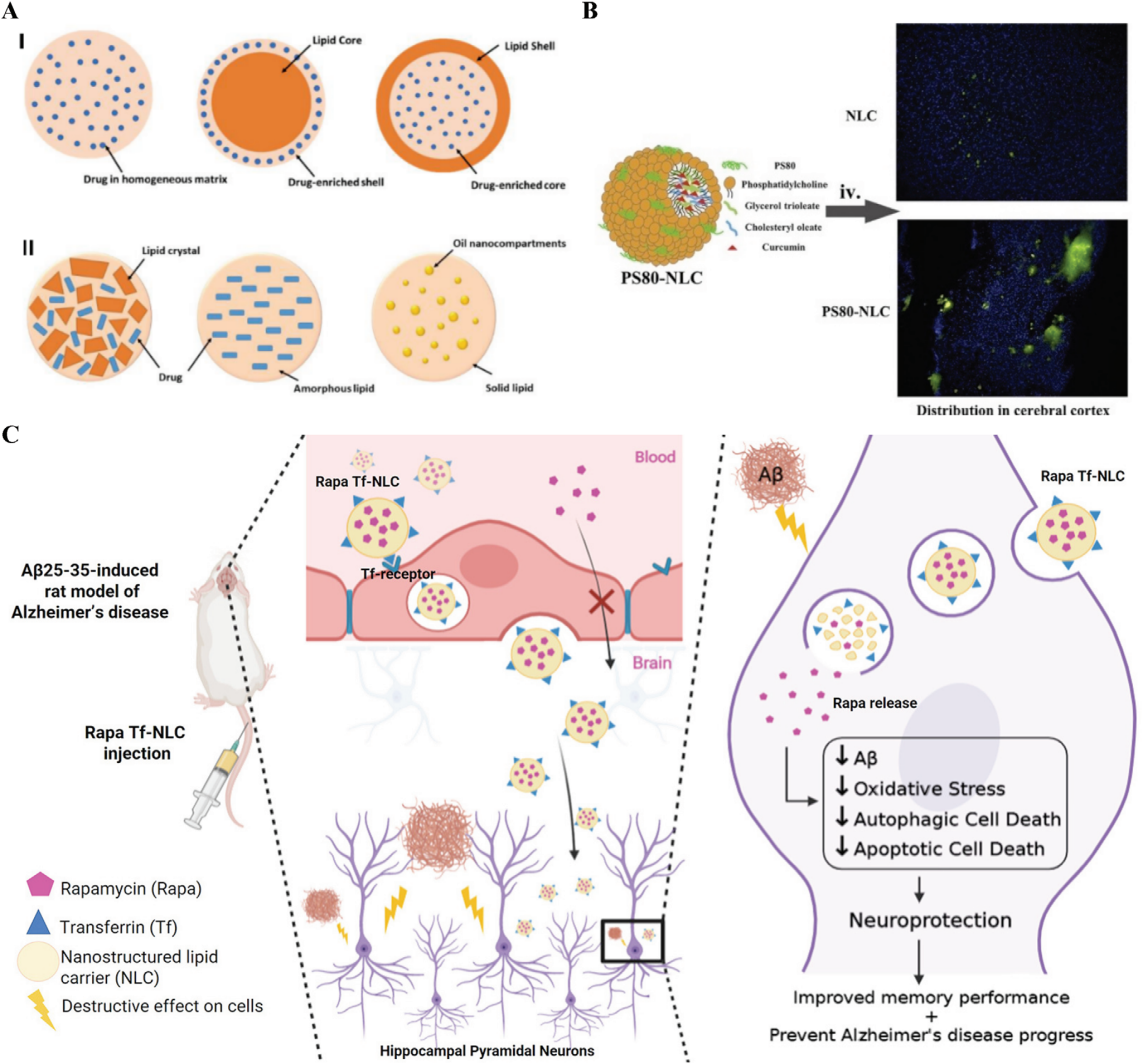
A) The drug incorporation models of SLNs (I): homogeneous matrix (a), drug‐enriched shell (b), and drug‐enriched core (c); and NLCs (II): imperfect crystal type (a), amorphous type (b), and multiple‐oil‐in‐fat‐in‐water type (c). Reproduced under the terms of the Creative Commons Attribution 4.0 International Public License.^[^ ^200^ ^]^ Copyright 2022, Chutoprapat et al. B) Lipoprotein resembling protein‐free Cur@NLC‐PS80 for brain targeting. Reproduced with permission.^[^ ^203^ ^]^ Copyright 2016, Elsevier. C) Tf‐NLCs are a promising delivery system for rapamycin in AD. Reproduced with permission.^[^ ^204^ ^]^ Copyright 2022, Elsevier.

NLCs, conceived as the subsequent generation of SLNs, were developed nearly a decade after the advent of SLNs to mitigate the complications engendered by them.^[^ ^173^ ^]^ NLCs, fabricated from a blend of solid–liquid lipids that remain in a solid state at ambient temperature, enable the coexistence of solid and liquid lipids within the lipid phase, resulting in structural rearrangements and a reduction in crystalline degree, as depicted in Figure 8A.^[^ ^196^ ^]^ This unique configuration not only precludes the expulsion of the drug from the matrix but also enhances the drug loading capacity and bolsters the long‐term physical and chemical stability.^[^ ^173^ ^]^ Therefore, as an improved version of SLNs, NLCs present enhanced loading efficiencies, demonstrate improved stability as well as prevention in drug expulsion during storage.^[^ ^197^, ^198^ ^]^ The incorporation of drugs into SLNs and NLCs can be described by three models, as elucidated in **Figure** **9**A.^[^ ^199^, ^200^ ^]^

Same as SLNs, NLCs can encapsulate existing therapeutic compounds for AD to enhance their bioavailability and penetration to brain. Berberine, an isoquinoline alkaloid, was loaded in the NLCs for AD rendering several modes of action such as AChE inhibition, monoamine oxidase inhibition, reduction of Aβ levels, antioxidative activity, and cholesterol‐lowering ability. Raju et al. demonstrated that berberine‐loaded NLCs have the potential of brain targeting and are effective in an animal model of AD.^[^ ^201^ ^]^ In 2015, Meng et al. designed a novel LDL‐mimic NLC modified with Lf (Lf‐mNLC) to loaded Cur for brain‐targeted delivery AD treatment.^[^ ^202^ ^]^ In this work, the use of the identical lipids that make up decent density proteins was novel, leading to the creation of an LDL‐mimic nanocarrier, which can bind LDLR on the BBB cells. Therefore, the Lf modification and LDL‐mimicking characteristic make NLCs easier to deliver drugs to the brain. In vitro studies demonstrated that Lf‐mNLC has a 1.5‐fold up than simple NLC. Ex vivo imaging studies revealed that Lf‐mNLC has a threefold higher uptake than plain NLC. Then in 2016, their group developed a protein‐free NLC to deliver Cur by surface coating with PS80 to absorb apolipoprotein onto the surface, resembling natural lipoprotein and leading their way into the brain by lipoprotein receptor‐mediated endocytosis (Figure 9B). Ex vivo imaging studies revealed that Cur@NLC‐PS80 could effectively permeate the BBB and preferentially accumulate in the brain (three times greater than that of Cur@NLC).^[^ ^203^ ^]^ In 2022, a Tf‐decorated NLC containing rapamycin (Rapa) was designed and developed for brain‐targeted delivery (Figure 9C).^[^ ^204^ ^]^ The results suggested that Tf‐decorated NLCs in comparison with bare NLCs, showed a significantly higher cellular uptake (97% vs 60%) after 2 h incubation and further an appropriate brain accumulation with lower uptake in untargeted tissue in mice. The in vivo study showed the Rapa Tf‐NLC significantly improved the neurobehavioral and spatial memory in the rat model of AD and critically reduced oxidative stress and neurotoxicity induced by Aβ compared to free rapamycin.^[^ ^204^ ^]^ Therefore, the Tf‐decorated NLCs is promising tool for AD therapy.

### 3.2 Polymer‐Based

#### 3.2.1 Polymeric Nanoparticles (PNPs)

PNPs are widely used to develop innovative drug delivery system in the treatment of neurodegenerative and brain associated diseases, including AD. PNPs provide protection to the drugs via encapsulating, entrapping them inside the core, and conjugating, or adsorbing them on to the particle surface.^[^ ^205^ ^]^ Over the past several decades, PNPs have been extensively synthesized and applied in brain drug delivery since they are able to be stable, easy to be prepared, biocompatible, biodegradable, nontoxic, sustained and extended drug release profile, minimal drug alteration, and allow brain‐targeted or site‐targeted delivery with modification.^[^ ^206^, ^207^, ^208^ ^]^ In particular, polymeric nanoparticles exhibit specific advantages compared to inorganic‐based nanosystems related to the opportunity to tune their physicochemical properties depending on the specific applications. The wide selection of starting materials (i.e., monomers or preformed polymers) and the synthesis protocols enable the modulation of charge, size, shape, surface area, encapsulation efficiency, and controlled drug delivery performances.^[^ ^209^ ^]^ The key parameters are related to the NP size, stiffness, shape, and to the ligand density. Both natural and synthetic polymers are employed to make up the brain‐targeting polymeric NPs with the size varying from 10 to 1000 nm. The natural polymers used for the polymeric NPs carriers are polysaccharides such as alginate, chitosan and hyaluronic acid and proteins including gelatin, collagen, fibrin, silk, and albumin. Synthetic polymers employed are biologically inert and hydrolytically degradable, safe, and exhibit low immunogenicity, which include poly(lactic acid) (PLA), poly(glycolic acid), poly(lactic‐*co*‐glycolic acid) (PLGA), and poly(butyl cyanoacrylate) (PBCA).^[^ ^210^ ^]^

Using PNPs to deliver therapeutic drugs in a tailored manner into the brain of AD patients is by far one of the most promising and cutting‐edge strategies. These smart nanosystems provide a novel and safe tool for increasing the efficacy of pharmaceuticals administered parenterally via improved pharmacokinetics and biodistribution.^[^ ^211^ ^]^ Some therapeutic compounds including donepezil,^[^ ^212^ ^]^ galantamine,^[^ ^213^ ^]^ rivastigmine,^[^ ^214^ ^]^ resveratrol,^[^ ^215^ ^]^ and quercetin,^[^ ^216^ ^]^ have been reported to be encapsulated into PNPs synthesized by various materials and methods for AD treatment in both in vitro and in vivo studies. However, these drug‐loaded PNPs still have many limitations especially the limited BBB penetration. To ease the BBB entry, several ligands can be conjugated to the surface of PNPs. These molecules are grouped into four different types: i) ligands that adsorb proteins from the bloodstream able to interact directly with receptors or transporters expressed on the endothelial compartment of the BBB, such as polysorbate 80;^[^ ^217^ ^]^ ii) ligands that directly bind BBB receptors or transporters, such as targeting ligands or Abs specific for the TfR, insulin receptor or glucose transporter;^[^ ^85^, ^87^, ^218^, ^219^, ^220^, ^221^ ^]^ iii) ligands capable of increasing charge and hydrophobicity, such as amphiphilic peptides;^[^ ^222^ ^]^ and iv) ligands able to enhance blood circulation time (e.g., PEG).^[^ ^223^ ^]^

Polysorbate 80‐coating PBCA NPs were the first polymeric NPs used for drug delivery across the BBB in 1995.^[^ ^224^ ^]^ It is due to the ability of polysorbate 80 to absorb plasma apolipoprotein E (Apo‐E) causing NPs coated with polysorbate 80 to be recognized as LDL. Then the coated PBCA NPs will be taken in by the brain ECs with the help of LDL receptor on these cells through a receptor‐mediated endocytosis route.^[^ ^225^, ^226^ ^]^ NGF, which is vital for central cholinergic neuron survival, adsorbed on PBCA NPs coated with polysorbate 80 was administered in mice and reached the brain parenchyma in significantly higher amounts at 45 min after administration.^[^ ^227^ ^]^ For the second case, Lf‐conjugated N‐trimethylated chitosan (TMC) was used to modify the surface of PLGA NPs to deliver Huperzine A (an AChE inhibitor) and then the carrier showed 2.5‐fold higher brain absorption compared with that of the nonmodified carrier.^[^ ^228^ ^]^ In the third case, the ligands assist the PNPs in crossing BBB via AMT mechanism. The TAT peptide conjugated on the surface of PLA NPs promoted an increase in transport of the same NPs through the BBB via the bypass of efflux transporters, suggesting that it is a promising tool for AD treatment. For the fourth case, as mentioned before, PEGylation provides higher stability to NPs, allows them to circulate for prolonged time and protects them from the immune system, thus enhancing biodistribution and effectiveness of drug loading.^[^ ^229^ ^]^ In work completed by Sánchez‐López et al., memantine (MEM), a drug approved for the treatment of AD, was loaded in PEG–PLGA NPs to target the BBB upon oral administration. MEM–PEG–PLGA NPs showed a slower release profile with respect to a free drug solution because of the ability to cross the BBB and the reduction of Aβ plaques.^[^ ^230^ ^]^

#### 3.2.2 Nanogels

**Figure 10:**
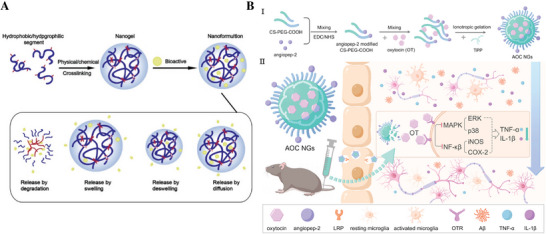
A) Representation of the synthesis and the release behavior of nanogels. Reproduced under the terms of the Creative Commons Attribution 4.0 International Public License.^[^ ^234^ ^]^ Copyright 2022, Zhang et al. B) AOC NGs inhibit innate inflammatory response for early intervention in AD. Reproduced with permission.^[^ ^238^ ^]^ Copyright 2022, American Chemical Society.

Nanogel was introduced in 1999 by Vinogradov et al. to describe the particles of a hydrophilic polymer network (PEG–PEI) obtained by crosslinking PEG and PEI that were able to deliver antisense oligonucleotides.^[^ ^231^ ^]^ These hydrogel nanoparticles are generally defined as 3D colloidal hydrogel nanoparticles obtained by physical or chemical crosslinking of the polymers with a diameter <200 nm, thus possessing both the advantages of a hydrogel and characteristics of a nanocarrier system (**Figure** **10**A).^[^ ^232^, ^233^ ^]^ Similar to the hydrogels, the hydrophilic groups within the polymeric structure of nanogels confer a high water retention capacity that enables absorption of large volumes of non‐immunoreactive liquids, thereby implying that nanogel formulations typically do not elicit an immune response. Given their swelling behavior and 3D crosslinked polymer network, various types of nanogels can deliver almost all types of therapeutic agents, including active biomacromolecules (such as DNA and siRNA), hydrophobic/hydrophilic drugs, proteins, vaccines, and even immunotherapeutics. Nanogels have unique advantages in CNS drug delivery, especially in increasing the penetration of the drugs through the BBB, enhancing the stability of the bioactive molecules against enzymatic degradation, and reducing the cytotoxic side effects. These unique advantages including: 1) stable physical/chemical properties; 2) high drug loading efficiency and efficacy for targeted delivery; 3) tunable size for crossing BBB; 4) straight forward surface modification/functionalization for stimuli‐responsive release and site‐specific drug delivery; and 5) biocompatible nanosized drug carriers and safety in brain. Therefore, nanogels are increasingly recognized as an advanced formulation of recently developed available drug carriers.^[^ ^234^ ^]^

Vinogradov et al. synthesized a new system based on a nanogel network of crosslinked PEG and PEI, which is capable of efficiently delivery of oligonucleotides (ODNs) to the brain across the BBB. The transport efficiency was further improved when the surface of nanogels was modified with Tf or insulin,^[^ ^235^ ^]^ suggesting nanogel is a promising system for delivery of ODN to the brain. Picone et al. developed an insulin loaded nanogels system as a new therapy for AD because of the protective role of insulin in AD. The results showed that insulin conjugated to nanogels could cross the BBB in mouse brain endothelial (bEnd3) cells and provide a neuroprotective effect against Aβ‐induced toxicity by reducing oxidative stress compared to administering “free” or nonconjugated.^[^ ^236^ ^]^ The development of the dual inhibitor nanosystems is expected to effectively target the inhibition of Aβ aggregation. Epigallocatechin‐3‐gallate (EGCG) and Cur can effectively inhibit Aβ aggregation. These two inhibitors were linked via a modification of hyaluronic acid, which induced 69% and 55% higher inhibition than EGCG‐ or EHA‐loaded single‐modified nanogels,^[^ ^237^ ^]^ respectively. In 2022, Ye et al. reported an oxytocin‐loaded angiopep‐2 modified chitosan nanogels (AOC NGs) that can cross the BBB through intravenous administration and enter the brain through angiopep‐2‐mediated transcytosis (Figure 10B). Angiopep‐2 is a 19 amino acid peptide that can bind LDLR expressed on BBB cells, and it had a higher transcytosis capacity and a better parenchymal accumulation than Tf, Lf, and avidin. Oxytocin (OT) is a highly conserved hypothalamic neuropeptide that exerts biological functions by specifically binding to its receptor (OTR), which is widely distributed throughout the brain. The results showed the AOC NGs can block the ERK/p38 MAPK and COX‐2/iNOS NF‐κB signaling pathways, showing its ability to effectively inhibit microglial activation and reduce inflammatory cytokine levels.^[^ ^238^ ^]^

#### 3.2.3 Micelles

**Figure 11:**
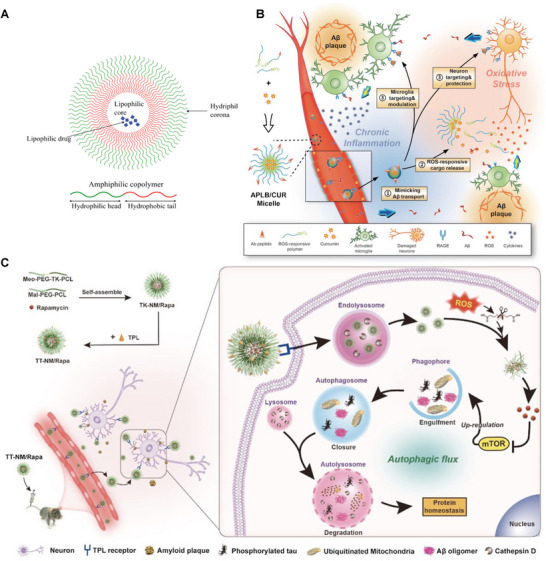
A) Representation of a polymeric micelle formed by an amphiphilic polymer with a lipophilic drug encapsulated in its lipophilic core. Reproduced under the terms of the Creative Commons Attribution 4.0 International Public License.^[^ ^97^ ^]^ Copyright 2022, Ayub et al. B) Illustration of microglia‐induced AD microenvironment and mechanisms of Ab–PEG–LysB/CUR modulation. Reproduced under the terms of the Creative Commons Attribution 4.0 International Public License.^[^ ^243^ ^]^ Copyright 2018, Lu et al. C) ROS‐responsive targeted micellar system (TT‐NM/Rapa) for improving neuronal proteostasis by upregulation of autophagic flux. Reproduced under the terms of the Creative Commons Attribution‐Noncommercial‐Noderivs 4.0 International Public License.^[^ ^244^ ^]^ Copyright 2022, Xu et al.

Comprised of amphiphilic polymers, micelles are distinguished by their distinctive spherical morphology, attributable to the segregation of hydrophobic sectors from hydrophilic counterparts, as shown in **Figure** **11**A.^[^ ^97^ ^]^ This segregation leads to the formation of an inner hydrophobic core surrounded by external hydrophilic terminals,^[^ ^239^ ^]^ a core–shell structure with size ranging from 10 to 100 nm. The inner hydrophobic core allows loading of hydrophobic drugs, while the external hydrophilic shell provides stability to micelles in an aqueous environment and prolongs their circulation time in bloodstream, thus protecting it from RES and promoting their accumulation in regions with permeable vasculature.^[^ ^240^ ^]^ Micellar drug delivery systems are popular owing to their small size, long circulation time, good stability, and targetability.^[^ ^241^ ^]^

The amphiphilic polymer can be synthesized via biomolecule grafting to yield micelles with augmented brain‐targeting efficacy. Agwa et al. prepared new natural self‐assembled micelles by covalently attaching Lf to conjugated linoleic acid (CLA) to load CLA once again to enhance its delivery to the CNS.^[^ ^242^ ^]^ CLA, a natural antioxidant, prevents the oligomerization and accumulation of Aβ peptides and phosphorylation of Tau protein. These drug‐loaded Lf‐CLA micelles demonstrated improved cognitive performance, diminished brain oxidative stress, inflammation, apoptosis, and acetylcholinesterase activity, alongside a reduction in Aβ_42_ deposition in an aluminum chloride‐induced AD animal model. Additionally, surface modification of micelles with targeting peptides can facilitate BBB penetration via AMT mechanism. Lu et al. proposed an ROS‐responsive polymeric micelle drug delivery system (Ab‐PEG‐LysB/CUR) with sequential targeting ability to normalize AD microenvironment via microglia modulation (Figure 11B).^[^ ^243^ ^]^ The targeting peptide (Ab), derived from Aβ protein, specifically binds to receptor for advanced glycation end‐products, which is overexpressed on BBB, neurons, and microglia cells in AD patients. Hence, the micelles can accumulate into the diseased regions and exert synergistic effects of polymer‐based ROS scavenging and cargo‐based Aβ inhibition upon microenvironment stimuli. In 2022, Xu et al. also designed an ROS‐responsive targeted micelle system (TT‐NM/Rapa) to enhance the delivery efficiency of rapamycin to neurons in AD lesions guided by the fusion peptide TPL, composed of a BBB‐penetrating peptide (TGN) and a GT1b ganglioside receptor‐binding peptide (Tet1) (Figure 11C).^[^ ^244^ ^]^ The system facilitates the intracellular release of rapamycin, an autophagy inducer, via ROS‐mediated disassembly of micelles. Consequently, it promotes the efficient clearance of intracellular neurotoxic proteins, Aβ and hyperphosphorylated Tau proteins, and ameliorates memory defects and neuronal damage in AD mice.

#### 3.2.4 Dendrimers

**Figure 12:**
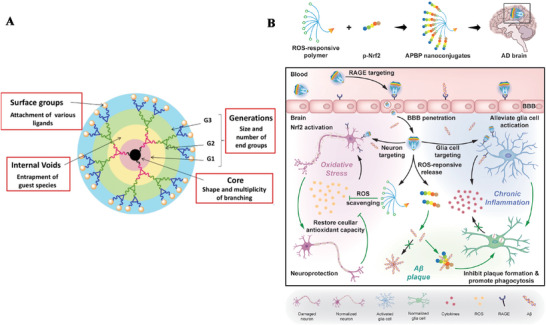
A) General features of dendrimer architecture. Reproduced under the terms of the Creative Commons Attribution 4.0 International Public License.^[^ ^246^ ^]^ Copyright 2015, Sharma et al. B) Illustration of APBP nanoconjugate formation and AD microenvironment modulation. Reproduced with permission.^[^ ^251^ ^]^ Copyright 2021, John Wiley and Sons.

Dendrimers, characterized by their highly branched, spherical polymeric architecture, have shown promise for use in treating AD. These polymers possess a unique 3D symmetry, incorporating an inner core and multiple hyperbranches, or “generations,” each terminating in various chemical functional groups. This structure allows for effortless functionalization with diverse ligands, as illustrated in **Figure** **12**A.^[^ ^245^, ^246^ ^]^ Dendrimer size, typically between 10 and 100 nm, is determined by the number of synthetic layers or “generations.”^[^ ^247^ ^]^ The extensive branching of dendrimers enables the conjugation of bioactive entities such as proteins or antibodies, making them appealing for targeted drug delivery. Moreover, peripheral groups contribute to crossing the BBB.^[^ ^248^ ^]^

Modification of the periphery of dendrimers provides a means of exploiting the different active pathways for crossing the BBB. Lf‐modified PAMAM dendrimers for delivering memantine to specific brain regions in AD‐induced mice via RMT has led to significant findings. Recent research has highlighted substantial effects on memory aspects in the target mice.^[^ ^249^ ^]^ Functionalization with a nutrient such as glucose, maltose, or amino acids such as histidine has been utilized to trigger the CMT mechanism to cross the BBB. Dendrimers with a poly(propylene imine) core and a maltose histidine shell (G4HisMal) have been successfully developed and may significantly alleviate AD symptoms, including memory dysfunction.^[^ ^250^ ^]^ Dendrimer–peptide conjugates have received significant attention for high drug loading content, easy modification, and good stability. In 2021, Liu et al. reported an ROS‐responsive dendrimer–peptide conjugate (APBP) to target the AD microenvironment and inhibit inflammatory responses at an early stage (Figure 12B).^[^ ^251^ ^]^ Herein, a targeting peptide (Ab peptide) is conjugated to a PEG‐based phenylboronic dendrimer for brain targeting and AD microenvironment accumulation via AMT, and then, a therapeutic peptide (p‐Nrf2) which can activate the nuclear factor (erythroid‐derived 2)‐like 2 (Nrf2) signaling pathway and restore the antioxidant ability of neurons, is also conjugated to the ROS‐responsive dendrimer for AD treatment. By eliminating ROS and releasing p‐Nrf2, this nanoconjugate has a synergistic effect of restoring cellular antioxidant capacity and alleviating glial cell activation. Inhibition of inflammatory responses and neuroprotective effects in the early stages of AD has been demonstrated in vitro and in vivo.

Dendrimers present advantages compared to other nanocarriers due to their branching structure's capacity to encapsulate large amounts of various drugs simultaneously. However, the high synthesis costs and the poorly understood long‐term toxicological effects of dendrimers warrant further study.

#### 3.2.5 Polymer‐Based Nanocomposites

The fabrication of polymeric nanocomposites has opened new horizons in the field of nanotechnologies, and their potential applications in the diagnosis and management of AD have been explored recently. These composites, defined as a synthesis of diverse materials wherein the matrix is polymer‐based and the dispersed phase comprises nanofillers, entities characterized by at least one dimension being less than 100 nm, serve not only to enhance the foundational properties of the parent polymer and preserve the processing characteristics akin to the original polymers, but also to endow the composite material with novel functionalities.^[^ ^252^ ^]^ Thus, owing to the integrated multicomponent nature, these nanocomposites inherently exhibit multifunctionality, rendering them particularly adept for addressing the complexities inherent in AD pathology.

**Figure 13:**
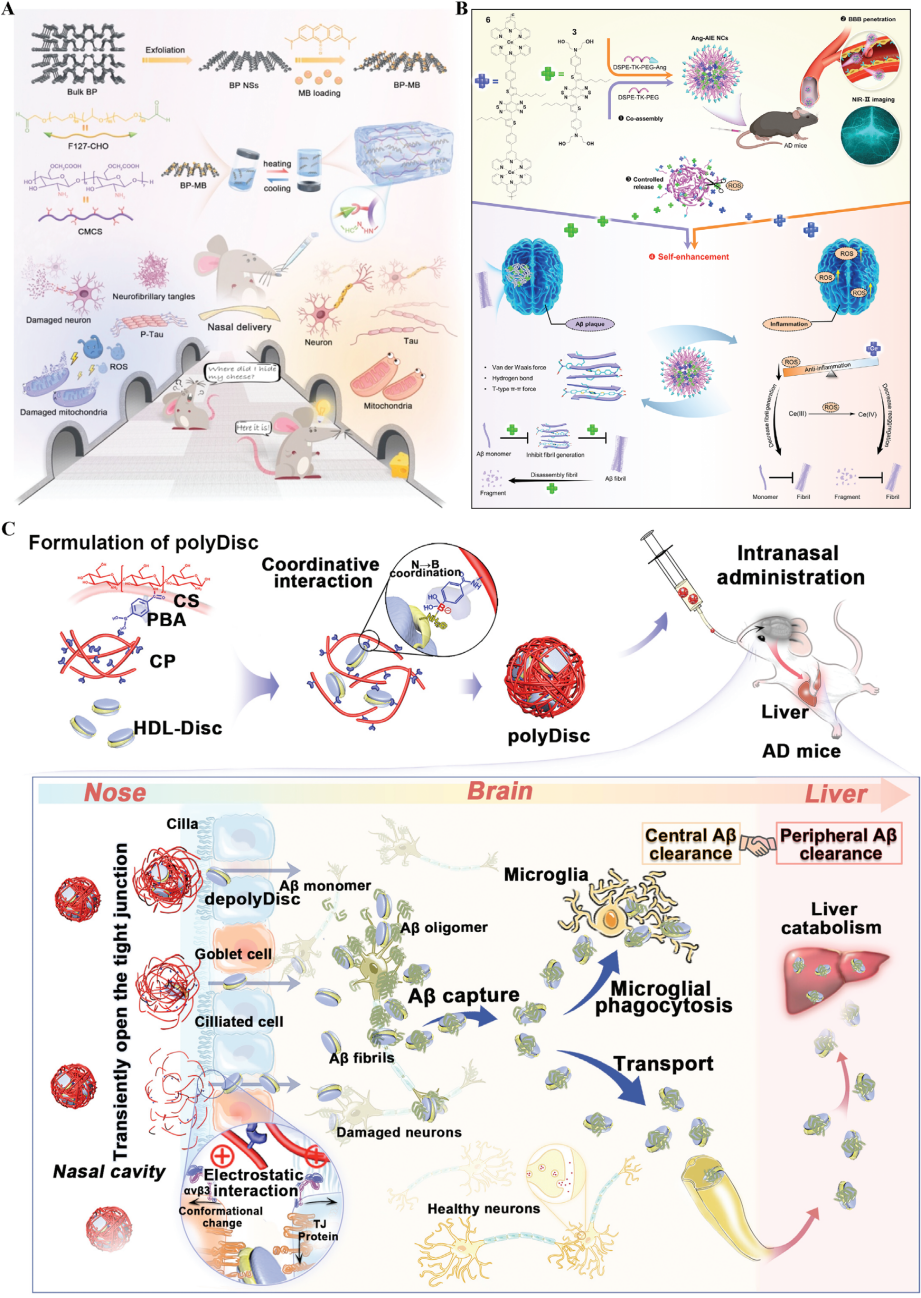
A) Schematic overview of the preparation of BP‐MB@Gel and its application for improving AD pathology. Reproduced with permission.^[^ ^253^ ^]^ Copyright 2024, John Wiley and Sons. B) NIR‐II brain‐target theranostic system for dual‐target therapy of AD. Reproduced under the terms of the Creative Commons Attribution 4.0 International Public License.^[^ ^254^ ^]^ Copyright 2024, Wang et al. C) PolyDisc preparation and transportation for Aβ systemic clearance in AD therapy. Reproduced under the terms of Creative Commons Attribution‐Noncommercial‐Noderivs 4.0 International Public License.^[^ ^255^ ^]^ Copyright 2023, Zhang et al.

Liu et al. recently reported an intranasal hydrogel‐delivering methylene blue (MB) system (BP‐MB@gel) for brain‐targeted AD therapy (**Figure** **13**A).^[^ ^253^ ^]^ In this study, MB, a widely studied drug proven to prevent Tau aggregation and attenuate Tau phosphorylation, is loaded onto black phosphorus nanosheets (BP NSs), serving as a drug carrier and antioxidant to obtain BP‐MB. Then, they fabricated a thermosensitive hydrogel to incorporate the BP‐MB for intranasal delivery, providing high nasal mucosal retention, controlled drug release, and BBB circumventing. These nanocomposites exert synergistic therapeutic effects by suppressing Tau neuropathology, restoring mitochondrial function, and alleviating neuroinflammation, thus inducing cognitive improvements in mouse models of AD. In 2024, Wang et al. employed an angiopep‐2 modified, ROS‐responsive polymer for coassembly with two innovatively synthesized NIR‐II aggregation‐induced emission (AIE) molecules to form the nanocomposites. This polymer facilitates BBB penetration enabled by angiopep‐2 modification through RMT. Upon ROS activation, the two encapsulated theranostic molecules are controllably released to activate a self‐enhanced therapy program. One specifically inhibits the Aβ fibrils formation, degrades Aβ fibrils, and relieves the ROS as well as inflammation. Another effectively scavenges ROS and inflammation to remodel the cerebral redox balance and enhances the above therapy effect, together reversing the neurotoxicity and achieving effective behavioral and cognitive improvements in the AD mice model (Figure 13B).^[^ ^254^ ^]^ Zhang et al. propose an approach utilizing Aβ‐antibody mimic discoidal high‐density lipoproteins (HDL‐Disc) and polymeric assembly (polyDisc) for nasal brain delivery to bypass the BBB (Figure 13C).^[^ ^255^ ^]^ HDL‐Disc, possessing Aβ high binding affinity, is employed to capture and then direct Aβ from central to peripheral catabolism, with desirable safety and translation potential. The pH‐sensitive polyDisc undergoes depolymerization in acidic nasal microenvironment, leaving the grafted chitosan polymer adhered to the mucosal layer to reversibly open tight junctions, which help the released HDL‐Disc penetrate the olfactory pathway into brain. This study elucidates a synergetic Aβ clearance strategy by simultaneously mobilizing central and peripheral clearance of Aβ in AD therapy from a systemic view, shifting the focus from central clearance to systemic Aβ clearance.

### 3.3 Metal‐Based Nanomaterials

**Figure 14:**
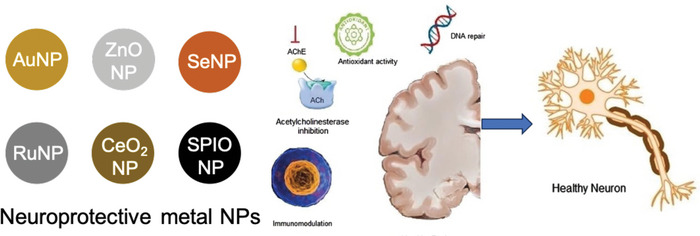
Neuroprotective mechanisms of metal‐based NPs affecting AD by modulating oxidative stress activity, acetylcholinesterase levels, and inflammation. Reproduced under the terms of the Creative Commons Attribution 4.0 International Public License.^[^ ^259^ ^]^ Copyright 2023, Anindita Behera et al.

Metal nanomaterials, mainly NPs, as well as their oxide NPs, exhibit unique mechanisms for applications in the diagnostics and treatment of AD, unraveling cancer biomolecules and biomarkers and nerve cells inflammation, as well as target drug delivery for various diseases.^[^ ^256^ ^]^ The preparation of NPs smaller than 100 nm significantly increases their surface area to volume ratio and enhances their ability to interact with organic and inorganic molecules for better drug delivery.^[^ ^257^ ^]^ These NPs can be supplemented through different routes of administration such as oral, intratracheal, intravenous, intraperitoneal, and intranasal administration.^[^ ^258^ ^]^ The enhanced bioavailability and/or efficacy associated with the formulation of targeted NPs of various drugs and bioactive agents used in AD treatment is a promising solution to overcome many of the existing challenges, as is illustrated in **Figure** **14**.

#### 3.3.1 Gold Nanoparticles

AuNPs have long been considered to play a major role among NPs in the field of AD.^[^ ^260^ ^]^ There has been considerable effort endeavored to probe the application of AuNPs in the field of AD diagnostics and treatment for their intriguing properties including tunable size, a large surface area‐to‐volume ratio, flexibility for versatile surface modifications, a high degree of biocompatibility, making them good candidates as carriers for target drug delivery across the BBB.^[^ ^261^ ^]^ AuNPs also possess antioxidant and anti‐inflammatory properties, which can be harnessed to induce neuronal activities for treatment of AD.^[^ ^262^ ^]^ It also promotes microglia polarization toward M2 phenotype and reduce mitosis of astrocytes, which is beneficial for neuronal repair.^[^ ^263^ ^]^

**Figure 15:**
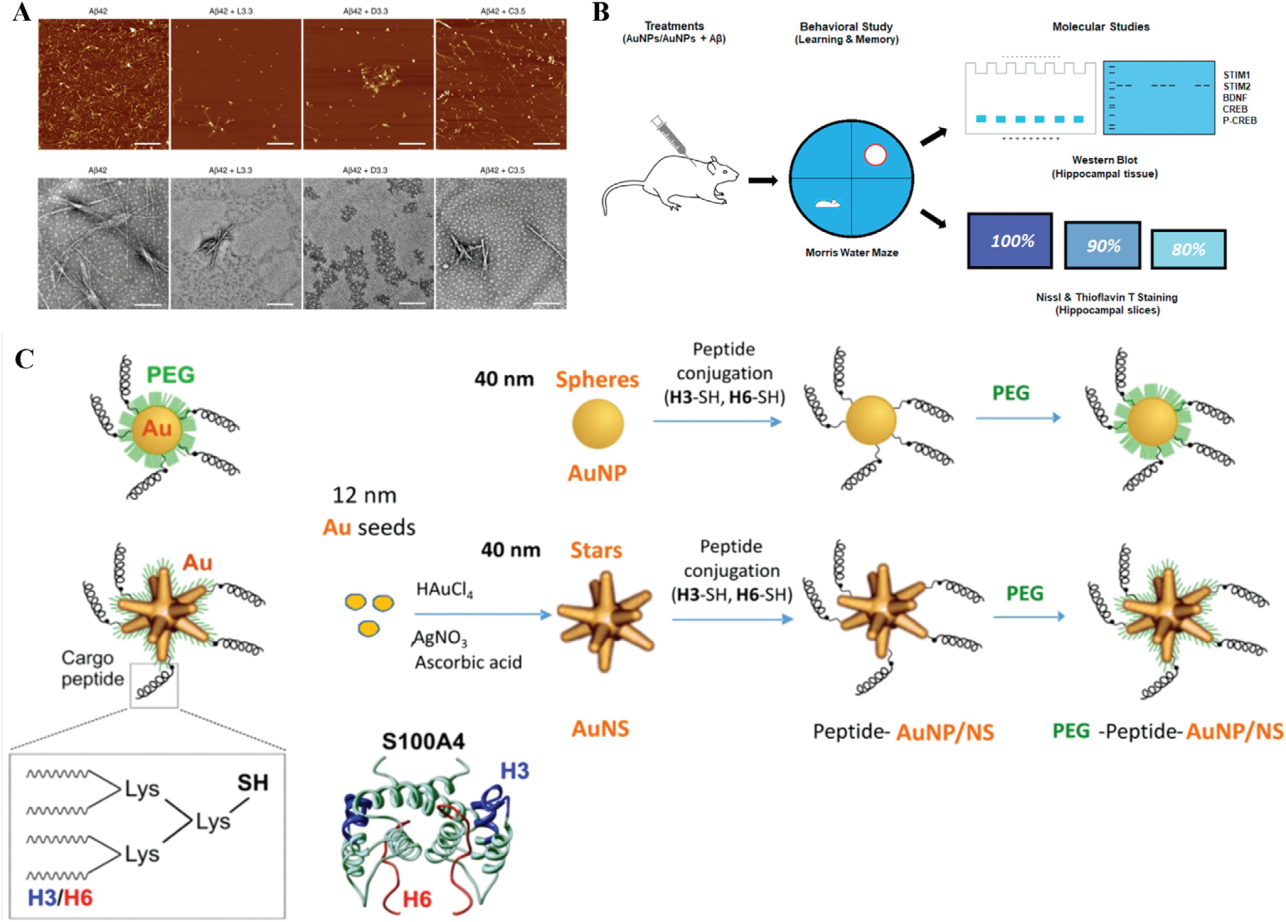
A) AFM images of Aβ_42_ in the absence and presence of _L_3.3, _D_3.3, or _C_3.5 after coincubation for 48 h (Scale bars: 1 µm.), and TEM images of Aβ_42_ in the absence and presence of _L_3.3, _D_3.3, or _C_3.5 after coincubation for 48 h (Scale bars: 200 nm). Reproduced under the Creative Commons Attribution 4.0 International Public License.^[^ ^264^ ^]^ Copyright 2020, Ke Hou et al. B) Illustration of the MWM experiment and the results. Reproduced with permission.^[^ ^265^ ^]^ Copyright 2019, American Chemical Society. C) Scheme of the synthesis of PEG‐ and peptide‐conjugated spherical and star‐shaped Au nanostructures (AuNP/AuNS). Reproduced under the terms of the Creative Commons Attribution 4.0 International Public License.^[^ ^267^ ^]^ Copyright 2022, Corinne Morfill et al.

In a recent work done by Hou et al, ultrasmall‐sized AuNPs (3.3 nm) were used as potential anti‐Aβ therapeutics.^[^ ^264^ ^]^ GSH is known to protect tissues from damage caused by ROS, and thus possesses the therapeutic potential in AD, and the peculiarity of this study is the surface functionalization, where two chiral forms of GSH, _L_ and _D_, were selected as enantiomers to form Au nanoplatforms denominated as _L_3.3 and _D_3.3, respectively. The strategy made it possible to enantio‐selectively inhibit the aggregation of Aβ in vitro, proved by the comparison with bare citrate‐coated AuNPs, as illustrated in **Figure** **15**A. Furthermore, AuNPs were demonstrated to successfully cross the BBB in mouse models and to act as a therapeutic agent to counteract AD. Notably, Sela et al. have demonstrated that AuNPs were found to be distributed uniformly in both hypothalamus and hippocampus, indicating there is no selective binding of AuNPs in these regions of brain.

In addition to the antiaggregation effect, AuNPs have also been demonstrate to be inducive to ameliorating side effects triggered by Aβ aggregation, that is, the loss of cognitive abilities. Sanati et al. investigated the learning/memory‐related behaviors and the biomarkers that indicate cognitive abilities in the hippocampus before and after injections of 5 nm AuNPs, which were small enough to cross the BBB.^[^ ^265^ ^]^ A comparison was made between bare NPs and bucladesine‐functionalized NPs in rat models (Figure 15B). The outcomes revealed that the AuNPs could improve the acquisition and retention of spatial learning and memory in Aβ treated rats as indicated by the probe tests in Morris water maze (MWM). Anthocyanins, found in fruits, grains, and flowers, are reported to have antioxidant, anti‐inflammatory, and antiapoptosis properties. Kim et al. used anthocyanins for encapsulation in PEG‐coated AuNPs to target the brain for Aβ clearance.^[^ ^266^ ^]^

Tuning the shape of AuNPs is likely to exert different effects in neuroprotection also with distinct molecule functionalization, in particular, the binding of dendrimers named H3/H6 on spherical and star‐shaped NPs in hippocampal neurons. These NPs were able to cross the BBB and induced the protection of neurons from oxidative stress and Aβ aggregation. Notably, the star‐shaped nanostructures induced greater effects than spherical NPs, suggested by the study of Morfill et al.^[^ ^267^ ^]^

**Figure 16:**
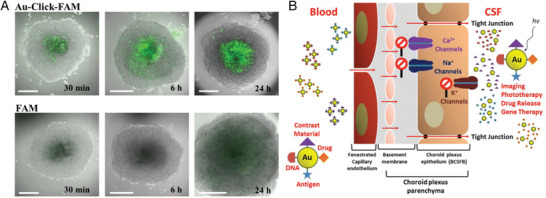
A) Uptake of ultrasmall FAM‐labeled AuNPs (Au‐Click‐FAM) and dissolved FAM by brain spheroids after 30 min, 6 h, and 24 h. Scale bar is 200 µm. Reproduced under the terms of the Creative Commons Attribution 4.0 International Public License.^[^ ^271^ ^]^ Copyright 2020, Viktoriya Sokolova et al. B) Schematic description of light‐triggered biomedical applications using functionalized AuNPs that can penetrate the BBB. Reproduced under the terms of the Creative Commons Attribution 4.0 International Public License.^[^ ^273^ ^]^ Copyright 2015, Hagit Sela et al.

With regards to the application of drug carriers of AuNPs, the size of AuNPs plays a critical role in its ability to permeate through BBB. Animal studies suggest that AuNPs in the sub‐100 nm range could enter the brain parenchyma after systemic administration.^[^ ^268^ ^]^ Betzer et al.^[^ ^269^ ^]^ synthesized insulin‐coated AuNPs with different sizes (20, 50, and 70 nm) and studied the biodistribution and their ability to cross the BBB in mice models, proving that 20 nm AuNPs exhibited the best BBB crossing ability among all. More significantly, when crossing the BBB, no damage to the barrier integrity of the BBB will be caused by AuNPs.^[^ ^270^ ^]^ Sokolova et al. probed the BBB crossing ability of AuNPs by comparing the FAM‐functionalized 2 nm AuNPs with the dissolved FAM, which was not able to cross the BBB (**Figure** **16**A).^[^ ^271^ ^]^ The ultrasmall AuNPs were able to enter the brain spheroid and accumulated in the center of the spheroid, indicating that ultrasmall AuNPs could preferentially cross the BBB.

Furthermore, while exploring the mechanism of ultrasmall AuNPs crossing the BBB, it was found that when ion channel blockers were employed, the penetration through the BBB was reduced to half compared to the control group, which suggests that the ion channel blockers could also control the penetration through the BBB, and that the ion channel blockers affected the AuNPs crossing the BBB by reducing the direct passage of AuNPs through the channel.^[^ ^272^ ^]^

**Figure 17:**
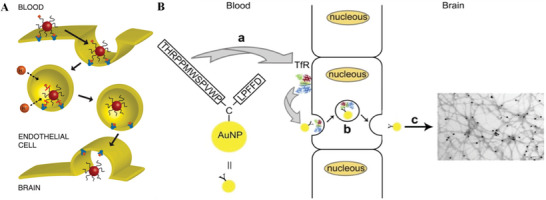
A) Mechanism of transcytosis for Tf‐containing NPs. Reproduced with permission.^[^ ^274^ ^]^ Copyright 2015, Andrew J. Clark et al. B) Scheme of the conjugate AuNP–THR–LPFFD. (a) The peptide THR–CLPFFD anchored to the AuNP. (b) transportation of the endocytic vesicle through the BBB. (c) Recognition and binding of the conjugate to Aβ aggregates inside the CNS. Reproduced with permission.^[^ ^276^ ^]^ Copyright 2012, Elsevier.

Based on the above evidence, it can be deduced that the BBB crossing ability of unmodified AuNPs is much limited. Therefore, more efforts had been put into the surface modification of AuNPs in this field. In another study, Clark and Davis prepared a Tf‐containing AuNPs (≈80 nm), which could be cleaved in acidic conditions (**Figure** **17**A).^[^ ^274^ ^]^ This design was used to investigate the mechanism of crossing the BBB via RMT based on the relation between TfR, and there was a threefold increase in the amount of AuNPs in the brain parenchyma compared with the AuNPs with noncleavable linkers. Olmedo et al. used 12 nm AuNPs conjugated to the amphiphilic breaker peptide LPFFD modified with a Cys residue (CLPFFD), to recognize Aβ and facilitate the penetration into the BBB.^[^ ^275^ ^]^ However, the percentage of AuNP–CLPFFD that reaches the brain is still below expectation. To this end, Prades et al. used a BBB shuttle to increase the permeability of the conjugate to the CNS by means of an active transport mechanism as shown in Figure 17B.^[^ ^276^ ^]^ The incorporation of TfR‐targeting peptide THRPPMWSPVWP (THR) was proved to be able to improve the delivery of the AuNP–CLPFFD conjugate to the brain via proactive targeting. These findings suggest that RMT can ameliorate the BBB crossing ability and that efforts on exploring more relevant and unique receptors beneficial for BBB crossing can be devoted.

Although most of the drug delivery based on NPs targets to pass the BBB directly with different routes, some effort has been made recently to use transnasal delivery to reach the BBB. In 2016, Zhang et al. synthesized a nanoconjugate consisting of WGA‐HRP, AuNPs, and drugs.^[^ ^277^ ^]^ WGA‐HRP was an enzyme that targets and penetrates into neurons. The conjugate was injected into the diaphragm of rats so that it could be delivered to the brainstem by retrograde transport directly to neuron cell bodies bypassing the BBB. Most importantly, compared with drug itself regarding the drug dosage and time required for the recovery of respiratory activity, the conjugate largely reduced the amount of drug and significantly extended the effective time of drugs. This significantly expands the application of AuNPs conjugate to the nanotherapy of AD avoiding BBB.

**Figure 18:**
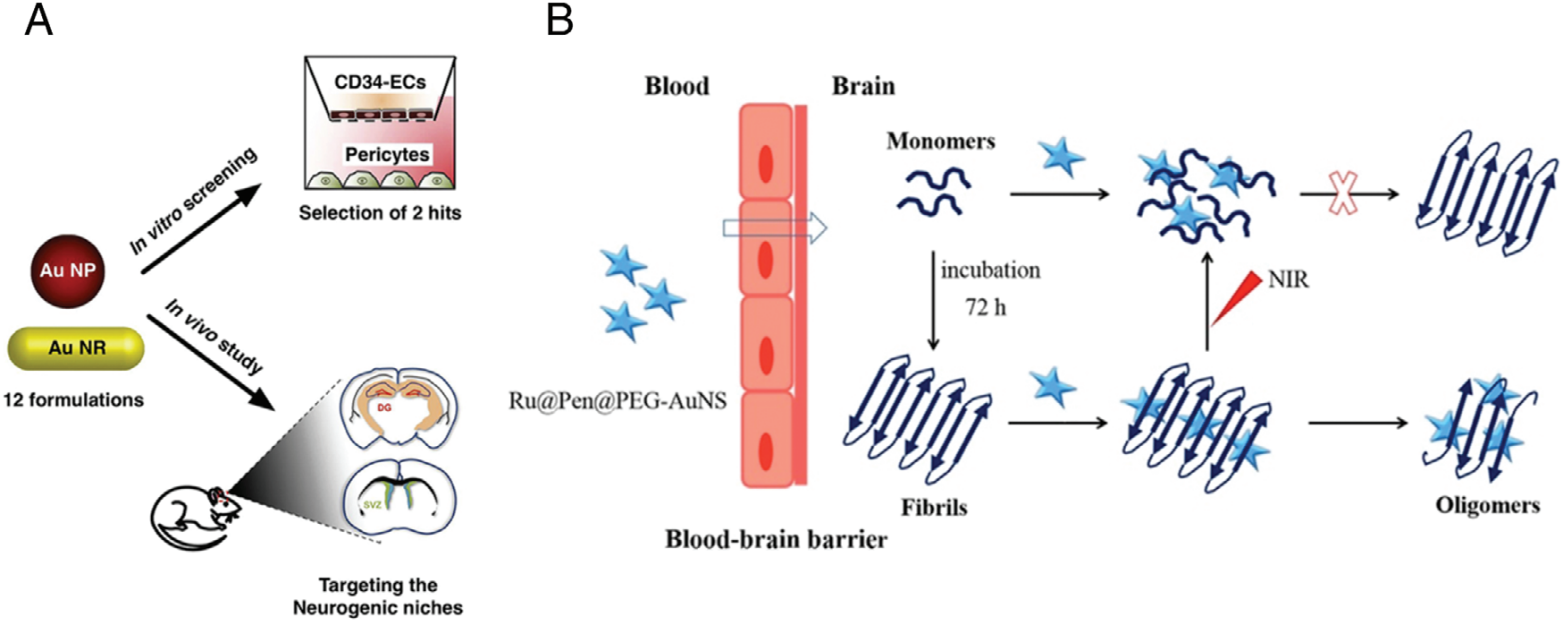
A) Scheme of methodology used to select NP formulations to target the neurogenic niches and BBB crossing. Reproduced with permission.^[^ ^278^ ^]^ Copyright 2018, Elsevier. B) Schematic illustration of therapeutic effect of Ru@Pen@PEG‐AuNS for Aβ fibrils dissociation with NIR irradiation. Reproduced with permission.^[^ ^279^ ^]^ Copyright 2016, American Chemical Society.

Although many kinds of surface functionalization of AuNPs to cross the BBB, assisting NPs cross the BBB with external force, such as NIR, still remains less explored. Praça et al. studied NIR laser exposure of the AuNPs/AuNRs and found that NIR could lead to the opening of the BBB due to a local heating effect (**Figure** **18**A).^[^ ^278^ ^]^ In vitro data showed that BBB integrity and permeability depend on the surface chemistry and concentration of the formulations along with NIR laser exposure. Compared with AuNPs‐Tf alone, the additional use of an NIR laser turned out the highest accumulation in the neurogenic niches, validating the use of photodynamic therapy (PDT) may offer further opportunities for the clinical translation of the approach in AD. NIR can also function as PDT in the field of AD treatment has been gaining more research interest recently. Penetratin (Pen) peptide has a powerful abilityn to conquer the poor BBB permeability and transmit cargoes without causing significant cytotoxicity, hence, Yin et al. synthesized PEG‐stabilized gold nanostars (AuNS) conjugated with Pen peptide (Pen@PEG‐AuNS), which show high permeability across the BBB (Figure 18B).^[^ ^279^ ^]^ Moreover, they add ruthenium (Ru(II)) complex that exhibits stable fluorescence properties to exert AD treatment under the presence of NIR.

AuNPs can be used not only as a vehicle for drug delivery but also as a drug, and more in‐depth studies are needed on the pathways of AuNPs to inhibit inflammation in AD models. Meanwhile, its biosafety is an issue of great concern, demanding further studies to find the right size as no definite conclusion has been made yet. The specific effects of AuNPs on AD and the cellular and molecular mechanisms entail further exploration in the years to come.

#### 3.3.2 Zn‐Based Nanoparticles

Zn‐based NPs with a wide diversity in nanostructures were found to be biosafe, nontoxic, biocompatible and prompted its use in numerous technologies and disease treatment, including AD. Distinctively, the U.S. FDA has classified ZnO as a generally safe compound.^[^ ^280^ ^]^ ZnO NPs have a remarkable potential in drug delivery, biosensing, biolabeling, and nanomedicines due to their anti‐inflammatory, anticancer, antiplasmodial, and antioxidant activities.^[^ ^281^ ^]^

AChE inhibition is an important target for the management of AD. El‐Hawwary et al. proved green‐synthesized ZnO NPs were able to decrease the level of AChE, which leads to reduced hydrolysis of the neurotransmitter acetylcholin and improved cognitive function.^[^ ^280^ ^]^

**Figure 19:**
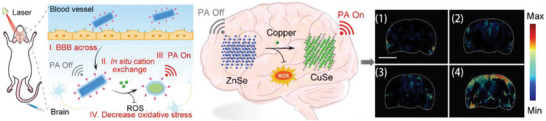
Schematic illustration of the in situ cation exchange reaction of Ang–ZnSe nanoplatelets with Cu^2+^ in vivo: (I) crossing the BBB, (II) succeeding in situ cation exchange reactions with Cu^2+^ (III) to activate PA imaging, and (IV) the resulting decrease of oxidative stress in the brain of AD mice. In vivo PA imaging of normal and AD mice was compared. Reproduced with permission.^[^ ^282^ ^]^ Copyright 2022, American Chemical Society.

The latest application of Zn for AD treatment was presented by Han et al., where they proposed the use of in situ cation exchange of zinc selenide (ZnSe) nanoplates with AD mice brain endogenous copper, generating copper selenide (CuSe) nanocrystals that exhibits specific PA signal as they efficiently convert NIR into heat (**Figure** **19**).^[^ ^282^ ^]^ To facilitate the transport of the nanoplatelets across the BBB, they elaborately tethered the nanoplatelet surface with Angiopep‐2 (Ang) peptide, a ligand targeting lipoprotein receptor‐related protein 1 (LRP1) overexpressed on the BBB. The resulting nanoplatelet probe could efficiently cross the BBB and rapidly exchange with endogenous copper ions, enabling activatable boosting of PA imaging of brain copper levels. The experiments demonstrated that the nanoplatelets could efficaciously inhibit ROS generation and protect neuronal cells from necrosis and apoptosis, highlighting its potential for gaining insights into the pathophysiology study of AD.

#### 3.3.3 Selenium Nanoparticles (SeNPs)

SeNPs have various effectual characteristics that display biomedical applications and preventive effects on AD. SeNPs display efficient antioxidant properties as they improve antioxidant defence mechanisms and bioenergetics and reduce oxidative stress. There is a correlation between increased cognitive decline with decreased selenium levels.^[^ ^283^ ^]^ The SeNPs perform an important mechanism for inhibiting ROS production and increasing neuronal longevity by regulating the oxidative defense system, inflammatory reactions, cellular metabolic state, and functional properties of hippocampal neurons.^[^ ^284^ ^]^ It is also reported that SeNPs have greater bioavailability and biological activity with decreased cytotoxicity. Moreover, SeNPs were proved to significantly decompose Aβ fibrils into nontoxic fibrils, reduce p‐Tau, and alleviate neuroinflammation, resulting in the downregulation of AD progression.^[^ ^285^ ^]^

**Figure 20:**
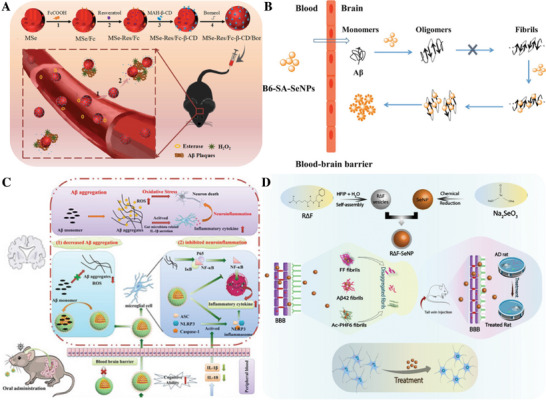
A) Schematic interpretation of Bor release and its penetration through the BBB. Reproduced with permission.^[^ ^288^ ^]^ Copyright 2019, Elsevier. B) B6‐SA‐SeNPs across BBB and Aβ disaggregation effects. Reproduced with permission.^[^ ^289^ ^]^ Copyright 2015, Elsevier. C) Aβ disaggregates and enhanced BBB crossing ability. Reproduced with permission.^[^ ^290^ ^]^ Copyright 2022, American Chemical Society. D) The ability of RΔF‐SeNPs to cross the BBB and the improvement of cognitive performance. Reproduced with permission.^[^ ^291^ ^]^ Copyright 2022, American Chemical Society.

**Figure 21:**
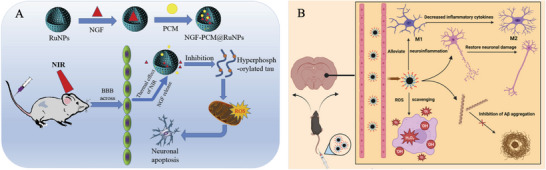
A) NGF‐PCM@Ru NPs cross the BBB under NIR irradiation. Reproduced with permission.^[^ ^297^ ^]^ Copyright 2019, Elsevier. B) Combined therapeutic mechanism of RuO_2_@Bor for AD treatment. Reproduced with permission.^[^ ^298^ ^]^ Copyright 2022, American Chemical Society.

In recent years, SeNPs have been gaining surging attention as a therapeutic moiety for the treatment of AD. Huo et al. designed and obtained a facile SeNP‐doped Cur‐PLGA polymer with the excellent size, morphology, and chemical structure for enhancing cellular uptake mechanism and increasing ability to transport into the BBB model.^[^ ^286^ ^]^ Yang et al. applied two targeting peptides (LPFFD and TGN) to conjugate to SeNPs, rendering the synthesized SeNPs able to cross the BBB and possess a strong affinity toward Aβ species, and thus, they can efficiently suppress extracellular Aβ fibrillation by disrupting hydrophobic and electrostatic interactions that are important for Aβ nucleation.^[^ ^287^ ^]^ Small molecules as surface modification are also applied to improve the BBB crossing ability of SeNPs. In a study conducted by Sun et al.,^[^ ^288^ ^]^ Borneol (Bor), a traditional Chinese medicine ingredient, that physiologically opens the BBB through neurotransmitters, was assembled into a hydrophilic‐sealed mesoporous nanoselenium (MSe) based nanodelivery system (Figure 21A).^[^ ^288^ ^]^ Bor was released under the hydrolysis action of blood or intracellular esterases to allow the nanosystem to accumulate rapidly in the brain. Sialic acid, an essential nutrient for optimal brain development and cognition, was modified on SeNPs conjugated with the peptide‐B6 (B6‐SA‐SeNPs) by Yin et al., which exhibited high permeability across the BBB and further inhibited as well as disaggregated Aβ fibrils in AD (**Figure** **20**B).^[^ ^289^ ^]^ Yang et al. coated SeNPs with dihydromyricetin (DMY), a natural product with anti‐inflammatory effect, to form DMY@SeNPs, which was further decorated with the BBB‐targeting peptide Tg (TGNYKALHPHNG), which diminished the aggregation of Aβ and enhanced the BBB crossing ability (Figure 20C).^[^ ^290^ ^]^ More recently, Kour et al. have utilized an amphipathic dipeptide, arginine‐dehydrophenylalanine (RΔF) derived nanospheres, as capping entities for the presynthesized SeNPs (Figure 20D).^[^ ^291^ ^]^ This coating enabled the noninvasive administration of SeNPs into brain tissues.

#### 3.3.4 Ruthenium Nanoparticles (RuNPs)

Among all inorganic transition metal nanomaterials, RuNPs have achieved an excellent profile due to their biocompatibility, large surface energy, and good packaging efficiency.^[^ ^292^ ^]^ RuNPs possess an inhibitory effect against p‐Tau and Tau aggregation, oxidative stress, and neuronal apoptosis and improve impaired learning and memory function.^[^ ^293^ ^]^ RuNPs are considered to be responsible for inhibiting Aβ fibrils, Aβ‐induced cytotoxicity, and enhancing neuronal outgrowth, suggesting that it can be used significantly in treating and preventing AD.^[^ ^294^ ^]^ It also suppresses Zn^2+^‐Aβ mediated generation of ROS, intracellular Aβ40 fibrillation, and their corresponding neurotoxicity in PC12 cells.^[^ ^295^ ^]^

Noticeably, RuNPs boast excellent photothermal effects under NIR irradiation,^[^ ^296^ ^]^ giving them the potential to increase BBB permeability and temperature‐responsive drug release. On this account, Zhou et al. first successfully prepared a novel flower‐like hollow RuNPs as a carrier, loaded with the therapeutic agent NGF (**Figure** **21**A), which effectively penetrated BBB, delivered NGF, and intelligently responded thermal effects to control NGF release under NIR irradiation, thereby achieving repair of neuronal damage and inhibiting multiple key pathways of p‐Tau‐related AD pathogenesis.^[^ ^297^ ^]^

Ruthenium dioxide (RuO_2_) is also applied for the treatment of AD. Tang et al. reported the RuO_2_@Bor with good biocompatibility and excellent antioxidant enzyme activity (Figure 21B).^[^ ^298^ ^]^ To overcome the obstruction of the BBB and improve the utilization efficiency of drugs, Bor, which physiologically opens BBB by affecting neurotransmitters, was leveraged. Results proved that RuO_2_@Bor could efficiently reduce the level of oxidative stress, inhibit Aβ aggregation, thereby reducing neuronal damage in AD mice.

#### 3.3.5 Cerium Dioxide Nanoparticles (CeO_2_/Ceria NPs)

CeO_2_ NPs have been reported to show protective effects against oxidative stress, decrease Aβ plaques development in cortical neurons via the regulation of mitochondria.^[^ ^299^ ^]^ In this respect, CeO_2_ NPs are gaining more attention and thus promising for preventing AD pathophysiology.^[^ ^300^ ^]^ Ultrasmall CeO_2_ NPs also boast the merits of control of neurotoxic cell signaling, and potent free‐radical‐neutralizing features in divergent tissues.

**Figure 22:**
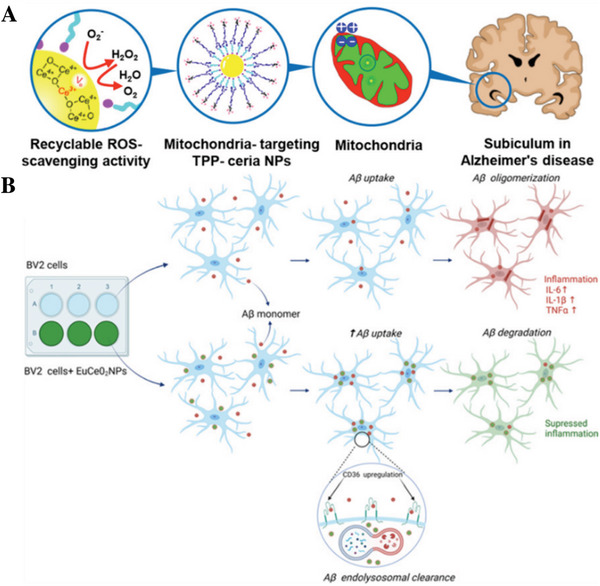
A) TPP‐Ceria NPs localize to mitochondria of subicular cells due to their small hydrodynamic diameter and ROS‐scavenging effect. Reproduced with permission.^[^ ^301^ ^]^ Copyright 2016, American Chemical Society. B) The illustration of the pathways of EuCeO_2_ NPs for AD treatment. Reproduced with permission.^[^ ^302^ ^]^ Copyright 2022, American Chemical Society.

Kwon et al. reported the design and synthesis of triphenylphosphonium‐conjugated ceria nanoparticles (TPP‐Ceria NPs) that localize to mitochondria and suppress neuronal death in AD mouse model (**Figure** **22**A).^[^ ^301^ ^]^ The synthesized TPP‐Ceria NPs mitigate the reactive gliosis and morphological mitochondria damage observed in the mouse model. By virtue of the ability of ultrasmall NPs to cross the BBB, TPP‐Ceria NPs are a potential therapeutic candidate for treating mitochondrial ROS‐induced damage in AD.

Machhi et al. assessed the immunomodulatory potential of europium (Eu)‐doped CeO_2_ NPs and verified that EuCeO_2_ NPs improved the microglial capabilities of Aβ phagocytosis and degradation (Figure 22B).^[^ ^302^ ^]^ By attenuating lipopolysaccharide (LPS)‐elicited proinflammatory microglial responses, EuCeO_2_ NPs were able to restore the homeostatic anti‐inflammatory and neuroprotective functions of microglia, indicating that EuCeO_2_ NPs could be developed as an AD immunomodulator.

#### 3.3.6 Superparamagnetic Iron Oxide Nanoparticles

Recent years has witnessed the vast application of SPIO NPs in targeted therapy, drug delivery, biological imaging, and other aspects, and are mainly characterized by good biocompatibility, superparamagnetic properties, high safety, and low toxicity. The most commonly used superparamagnetic nanoparticles are SPIO nanoparticles.^[^ ^303^ ^]^ SPIO NPs potentially interact with lysozyme amyloids, leading to a reduction of Aβ aggregates.^[^ ^304^ ^]^ The antiaggregating action of SPIO NPs was reported due to the adsorption of lysozyme onto the NPs and could be the possible reason for their anti‐AD effect.^[^ ^305^ ^]^

Wang et al. synthesized SPIO NPs labeled with human umbilical cord mesenchymal stem cells (hUC‐MSCs), which can significantly improve the targeting ability.^[^ ^306^ ^]^ hUC‐MSCs have the characteristics of differentiation, autologous transplantation, injury repair, and amplification. In order to further fortify the magnetic targeting, dopamine (PDA) was coated onto the surface of SPIO NPs. Compared with ordinary SPIO NPs, the SPIO@PDA NPs were more stable, and magnetic separation, targeting as well as MRI contrast were also improved. Quercetin was found to be able to ameliorate neurological diseases, yet unable to cross the BBB. Ebrahimpour et al. studied quercetin‐conjugated SPIO NPs against diabetes‐related learning and memory impairment in rats, and the results proved significantly enhanced BBB crossing capability and much recovered diabetes‐related memory impairment.^[^ ^307^ ^]^

**Figure 23:**
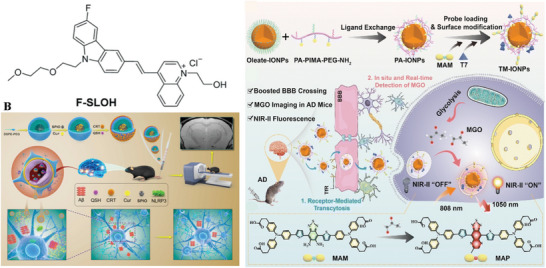
A) Chemical structure of F‐SLOH. Reproduced with permission.^[^ ^308^ ^]^ Copyright 2018, Wiley. B) Synthesis of SDP@Cur‐CRT/QSH and Aβ plaques targeting via MRI. Reproduced under the terms of the Creative Commons Attribution 4.0 International Public License.^[^ ^309^ ^]^ Copyright 2022, Yuting Ruan et al. C) Scheme of the preparation of TM‐IONPs and the nanoprobe passing through the BBB to visualize MGO in the brains AD mice. Reproduced under the terms of the Creative Commons Attribution 4.0 International Public License.^[^ ^310^ ^]^ Copyright 2022, Yi Lai et al.

Besides playing a therapeutic role in the field of AD treatment, it is also worth noting that FDA has already authorized SPIO NPs as MRI contrast agents in clinical imaging as SPIO NPs influence T_2_ relaxation times of surrounding tissues. Yet for AD imaging, a major clinical concern is the targeting of contrast agents to Aβ plaques in the brain. This limitation can be solved by linking peptides, immune targeting, imaging, and other targeting moieties to SPIO NPs. Li et al. developed a dual‐mode SPIO NP by conjugating a carbazole‐based oligomer‐selective cyanine probe, which showed strong affinity to Aβ in AD mouse models, with a slower signal decline and hypointensities corresponding to Aβ in high‐resolution in vitro MRI (**Figure** **23**A).^[^ ^308^ ^]^ Ruan et al. developed a novel curcumin‐conjugated SPIO NPs, SDP@Cur‐CRT/QSH, that efficiently pass the BBB. CRT, an iron peptide mimic, can bind to Tf for efficient BBB penetration, while curcumin can target Aβ plaques, and induce neuroprotection and neurogenesis (Figure 23B).^[^ ^309^ ^]^ Lai et al. designed the probe molecule for early AD diagnosis (Figure 23C).^[^ ^310^ ^]^ Peptide T7 worked as the targeting molecule for helping TM‐IONPs to penetrate BBB and accumulate into lesions. MAM molecules on the NP as NIR contrast agents with 1050 nm emission can respond to the biomarkers methylglyoxal (MGO) of AD.

### 3.4 Silica Nanoparticles (SiO_2_ NPs)

Thanks to their superior physiochemical properties such as large porosity, high surface areas, low toxicity, controllable sizes, and wide range of morphologies compared to conventional NPs, SiO_2_ NPs, especially mesoporous silica nanoparticles (MSNs) have emerged as favorable tools in biomedical applications as nanocarriers for encapsulation and delivery of therapeutic medicines.^[^ ^311^, ^312^, ^313^ ^]^ Designing biocompatible MSNs and their multifunctional derivatives for drug transport as well as theranostics has been one of the hottest areas of research in the field of nanobiotechnology and nanomedicine,^[^ ^314^ ^]^ and is auspicious for detection, diagnosis, and treatment of neurodegenerative disorders including AD.^[^ ^315^ ^]^

MSNs have been demonstrated to cross the BBB via AMT.^[^ ^316^ ^]^ To enhance the BBB uptake, various efforts have been made to initiate RMT. In 2016, Baghirov et al. investigated the uptake, transport, and cytotoxicity of MSNs as drug nanocarriers that were functionalized with PEG–PEI copolymer.^[^ ^317^ ^]^ The transport studies revealed that nonfunctionalized MSNs bore low permeability, and the cellular uptake experiments showed enhanced uptake of PEG–PEI‐functionalized MSNs. Neither bare MSNs, nor functionalized MSNs showed significant toxic effects on the cell viability indicating their potential safety for therapeutic purposes. Although the shape effect was negligible, but uptake studies observed using real‐time surface plasmon resonance (SPR) measurements, indicated that PEG–PEI coating of copolymers significantly contributed to enhanced uptake of MSNs, especially in case of rod‐shaped particles.

Previously, it has been noted that PTT can safely and reversibly open BBB by controlling light intensity to deliver more drugs to brain for therapy.^[^ ^318^ ^]^ The mesoporous silica‐encapsulated gold nanorods (MSNs‐AuNRs) with anti‐PD agent quercetin were prepared by Liu et al.^[^ ^319^ ^]^ Under 1064 nm laser irradiation  BBB permeability was fortified and the release of quercetin was ameliorated significantly.

The abundant receptors existing on the ECs membranes make RMT an outstanding option for crossing the BBB. To this end, Song et al. demonstrated a drug delivery system to the brain by conjugating Lf molecules on the surface of SiO_2_ NPs (PSi NPs). They observed relocation of functionalized NPs from the apical side to the basolateral side according to the size of particles, indicating size dependent high transport efficiency of Lf‐functionalized particles across the BBB. This RMT of silica NPs trough the ECs exhibits promising future for delivering therapeutic and imaging agents to the brain.^[^ ^320^ ^]^

**Figure 24:**
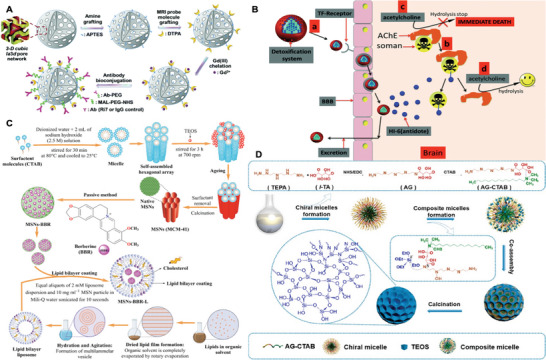
A) Functionalization steps for DTPA‐Gd and antibody grafting. Reproduced with permission.^[^ ^321^ ^]^ Copyright 2017, Royal Society of Chemistry. B) TF‐MSNs approach and cross the BBB, the drug is released, and TF‐MSNs are excreted. Reproduced with permission.^[^ ^323^ ^]^ Copyright 2016, Royal Society of Chemistry. C) Schematic presentation of BBR‐loaded MSN synthesis. Reproduced with permission.^[^ ^324^ ^]^ Copyright 2021, American Chemical Society. D) Schematic illustration for the self‐assembly synthesis of the *l*‐mSiO_2_ nanosphere. Reproduced with permission.^[^ ^326^ ^]^ Copyright 2023, American Chemical Society.

To study the effects of size and bioconjugation of MSNs on their targeting ability toward the brain microvessel ECs, Bouchoucha et al. reported the receptor‐mediated uptake of antibody‐conjugated MSN‐based nanocarriers by brain microvessel ECs (**Figure** **24**A).^[^ ^321^ ^]^ MSNs were functionalized with rat Ri7 antibodies, MRI contrast agent gadolinium chelate, and a fluorescent moiety. Cells incubated with Ri7‐MSNs (Gd incorporated) exhibited prominent MRI positive contrast enhancement signals, indicating their use as promising materials for theranostic applications. The particles accumulated in the brain microvessel ECs demonstrated strong potential of antibody‐treated MSNs for BBB penetration and brain ECs targeting. Recently, Wang et al. designed a novel nanoscale chelator SiO_2_–cyclen, which conjugated SiO_2_ NPs as delivery carriers with cyclen as a metal chelator, for inhibiting the metal‐induced Aβ toxicity.^[^ ^322^ ^]^ Outcome manifested that the amount of silica in mice brain increased nearly two times after 6 h, thus suggesting the nanochelator was able to penetrate the BBB. Yang et al. developed a detoxification system (DS) consisting of MSNs modified with Tf and loaded with the reactivator, HI‐6, which was made capable of effectively crossing the BBB via RMT (Figure 24B).^[^ ^323^ ^]^ To increase the lipophilicity of MSNs is another method to hone the BBB crossing ability. Therefore, Singh et al. leveraged lipid‐coated MSNs for the controlled and targeted delivery of berberine (BBR), a natural product conducive to AD treatment, to improve the BBB crossing ability (Figure 24C).^[^ ^324^ ^]^ Basharzad et al. reported a facile strategy to prepare highly biocompatible calcium‐doped MSN (MSN‐Ca) functionalized with polysorbate‐80 (PS) as a targeting ligand to deliver RV, an AD treatment molecule, to cross the BBB for AD treatment.^[^ ^325^ ^]^ Emerging evidence indicates that chiral inhibitors that bind to the α/β discordant stretch of Aβ_42_ peptides may exhibit enantioselectivity on inhibition of Aβ_42_ aggregation and is a key toward inhibiting Aβ_42_ peptides. Chiral helicates are potential therapeutic agents for enantioselectively inhibiting Aβ_42_ aggregation. Xu et al. thus put forward a chiral amide‐gel‐directed synthesis approach for preparing chiral mSiO_2_ nanospheres with molecular‐scale‐like chirality in the silicate skeleton (Figure 24D).^[^ ^326^ ^]^

Notably, the chiral mSiO_2_ specifically targets the Aβ_42_ central hydrophobic α/β‐discordant stretch, inhibiting the formation of β‐sheet structures and hence the fibrils. This new structure of MSNs is promising to provide novel platform for the coming surface functionalization to equip MSNs with the enhanced ability to cross the BBB.

MSNs have been extensively investigated as a viable drug delivery platform for treating AD owing to their high surface area‐to‐volume ratio, tunable pore size, and ability to traverse the blood–brain barrier effectively. Furthermore, MSNs were observed to act as nanoscavengers and have been proposed as a potential therapy for these neurodegenerative disorders as they can bind and remove toxic aggregates from the brain. However, it is important to note that while these studies have yielded promising results, further research is required to fully elucidate the potential safety risks.

### 3.5 Carbon Nanoparticles (CNs)

Small size of CNs permits BBB penetration and multiple surface functionalizations. They also allow easy conjugation or loading of molecules, and thus can be used to deliver drugs and chemicals into the brain, which is conducive to the treatment of neurodegenerative disorders such as AD.

The ultrasmall size and the versatile surface functionalities of carbon nanodots (CNDs) make them ideal candidates for the drug carriers of larger molecules through covalent conjugation. Wang et al. coated CNs with polymer that can be labeled with cell targeting agent interleukin‐6 (IL‐6).^[^ ^327^ ^]^ Experiment results validated the BBB penetration of the nanoconjugate.

Carbon nanotubes (CNTs) are needle‐like tubes and have good penetration capacity and large surface area, yet the major strike against CNTs is their hydrophobicity and toxicity, which can be overcome by surface functionalization. CNTs are manifesting promising future in the treatment of AD. Many AD therapeutic agents have been loaded inside the CNTs and targeted to brain cells and tissues by conjugating the surface with targeting molecules. Costa et al. functionalized MWCNT by conjugation with gadolinium L2, an Aβ targeting agent, which crossed the BBB via RMT and a therapeutic effect was achieved.^[^ ^328^ ^]^

**Figure 25:**
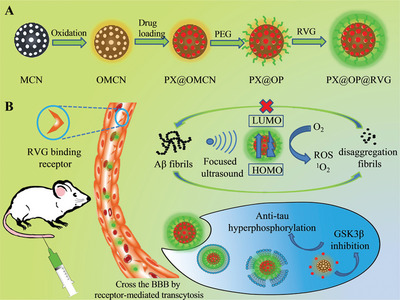
(I) Schematic illustration of the fabrication of the PX@OP@RVG NP. (II) NPs cross the BBB by RMT and double‐targeted treating process. Reproduced with permission.^[^ ^331^ ^]^ Copyright 2018, American Chemical Society.

Mesoporous carbon nanoparticles (MCNs) have been widely developed as a carrier because of its suitable particle size, good biocompatibility, and high drug‐loading capacity.^[^ ^329^ ^]^ Oxidized MCNs (OMCNs) have relatively higher water solubility and biocompatibility than MCNs, as well as an excellent photothermal effect, and can improve the permeability of the BBB of NPs during NIR irradiation.^[^ ^330^ ^]^ Protoporphyrin IX (PX) can result in the accumulation of ROS when used in the presence of ultrasound treatment, yet cannot cross the BBB. In this respect, Xu et al.^[^ ^331^ ^]^ harnessed the oxidized mesoporous carbon nanosphere (OMCNs) with high water solubility and biocompatibility, as well as an excellent photothermal effect, and can improve the permeability of the BBB. The conjugation with the brain targeting rabies virus glycoprotein (RVG) peptide increased the targeting ability of drugs to a specific site in the brain (**Figure** **25**). Experimental outcome proved the efficacy of the synthesized PX@OP@RVG on lowering the p‐Tau and on the inhibition of Aβ aggregation in the presence of focused ultrasound. This reported OMCN‐based NP not merely displays robust biocompatibility and brain bioavailability, but also permits the dual‐targeting therapy of AD. Its excellent photothermal effect provides a new way to improve BBB penetration.

CNs can pass through the BBB and enter the brain; thus, they can promise inspiring chances in the management of brain and neurodegenerative disorders. Therapeutic effects can be achieved by delivering medicines through CNs. The biocompatibility and cytotoxicity studies state that they are suitable for biological applications through various functionalizations. Newly discovered structures like carbon nanodiamonds and graphene‐related compounds have neuro‐regenerative activity, and they can be the future medicines for neurodegenerative disorders. However, further studies are required to find out their unrevealed activities in brain and neurons, which can open new treatment strategies against brain disorders.

### 3.6 Newly Emerging Nanomaterials

Cell membrane‐coated and metal–organic frameworks (MOFs)‐based nanoparticles are two recent innovations that ushered in the exploration of new nanosystems. Cell membrane‐coated nanoparticles, wrapped with biological membranes, utilize the natural properties of the membrane to achieve targeted delivery, reduced immunogenicity, and improved biocompatibility.^[^ ^112^, ^113^ ^]^ While MOFs are crystalline materials formed by the coordination of metal ions or clusters with organic ligands, offer a unique porous structure ideal for drug encapsulation and controlled release.^[^ ^332^ ^]^ Both nanoparticle systems are at the forefront of nanomedicine innovations, with the potential to enhance therapeutic efficacy and lead the next generation of drug delivery devices.^[^ ^333^, ^334^, ^335^, ^336^, ^337^, ^338^, ^339^ ^]^

#### 3.6.1 Cell Membrane‐Coated Nanoparticles

**Figure 26:**
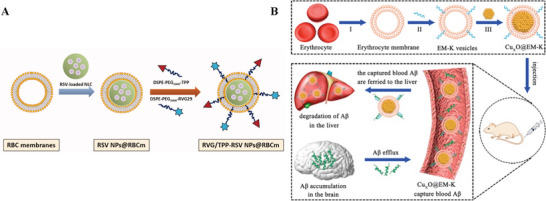
Cell membrane‐coated nanoparticles for AD treatment. A) Schematic preparation of RVG/TPP‐RSV NPs@RBCm. Reproduced under the terms of the Creative Commons Attribution 4.0 International Public License.^[^ ^104^ ^]^ Copyright 2020, Yang Han et al. B) Schematic diagrams of CuxO@EM‐K Synthesis and Peripheral Aβ Clearance by CuxO@EM‐K. Reproduced with permission.^[^ ^342^ ^]^ Copyright 2020, American Chemical Society.

In recent years, cell membrane biomimetic NPs that combine the uniqueness of natural cell membranes and an artificial nanoparticle core offer advantages for brain drug delivery. This kind of delivery system specifically extends the blood circulation time and bypass the immune system.^[^ ^109^ ^]^ For example, glycans, CD47, and sialic acid moieties on the surface of RBCs prolong the circulation time and reduce the immunogenicity of nanoparticles.^[^ ^110^, ^340^ ^]^ Many drug delivery systems have been proposed, utilizing membrane coatings derived from various cell sources, including RBC membrane, brain tumor cell membrane, immune cell membrane, and so on.^[^ ^341^ ^]^ RBCs are the most commonly used for erythrocyte membrane‐based biomimetic vehicles to improve the delivery for efficient CNS targeting. Recently, Zhang et al. constructed a nanoerythrocyte (NE) coating biomimetic delivery system, whose half‐life increased ≈2.5 times, compared with conventional liposomes. The prepared NE@DOX‐Ang2 drug carrier has features of favorable biocompatibility and low immunogenicity, and may provide a novel perspective for the development of clinically available nanomedicines using membrane‐coated nanoparticles.^[^ ^103^ ^]^ In another study by Han et al., a dual‐modified biomimetic nanosystem (RVG/TPP‐RSV NPs@RBCm) was constructed as shown in **Figure** **26**A to coat the NLCs, which are loaded with resveratrol (RSV) as a model antioxidant. The rabies virus glycoprotein (RVG29) and TPP molecules are attached to the RBC membrane surface. They found that RVG29‐modified nanoparticles showed an increased ability to penetrate the BBB in both in vitro coculture models and in vivo imaging studies. The therapeutic effect in vivo has been investigated as well. With the dual modifications, RVG/TPP NPs@RBCm can safely and specifically deliver antioxidants into neuronal mitochondria upon intravenous administration. The AD symptoms were relieved by mitigating Aβ‐related mitochondrial oxidative stress and the memory impairment in ADD mice was significantly improved.^[^ ^104^ ^]^

Recent studies have demonstrated that peripheral organs play a paramount role in the removal of Aβ from the brain. Clearance of peripheral Aβ can prominently facilitate a large efflux of Aβ from the brain into the blood through the sink effect and result in expeditious lowering of brain Aβ levels. Recently, Ma et al. designed an erythrocyte membrane decorated self‐protected biomimetic nanozyme (CuxO@EM‐K) to clean peripheral Aβ.^[^ ^342^ ^]^ CuxO nanoparticles with nanozyme activity were wrapped with KLVFF‐modified erythrocyte membrane for Aβ capturing (Figure 26B), which prevented the formation of undesirable protein coronas on the nanozyme with prolonged blood circulation time. The authors showed that CuxO@EM‐K could recognize and capture Aβ in blood, resulting in the alleviated Aβ burden.^[^ ^342^ ^]^ This work provides an example for clearance of AD‐related peripheral Aβ, which would be promising for overcoming the BBB hurdle to eliminate brain‐derived Aβ associated with AD.

Apart from commonly used erythrocyte membranes, immune cell membranes also offer unparalleled advantages for drug delivery.^[^ ^111^ ^]^ Immune cell surface proteins confer a variety of functionalities to NPs, such as extended circulation in the bloodstream, remarkable competency in recognizing antigens for enhanced targeting, enhanced cell interactions, and diminished in vivo toxicity.^[^ ^111^ ^]^ The immune system is composed of a diverse array of cells that work in concert to defend the body against pathogens including white blood cells, neutrophils, macrophages, natural killer cells, and so on.^[^ ^343^ ^]^ Their membranes are a promising choice for constructing drug delivery systems.^[^ ^111^ ^]^ Han et al. developed macrophage (MA) membrane‐coated solid lipid nanoparticles (SLNs).^[^ ^344^ ^]^ Rabies virus glycoprotein (RVG29) for crossing BBB and TPP molecules for targeting mitochondria of neurons was attached to the surface of MA membrane (RVG/TPP‐MASLNs) for the antioxidant Genistein (GS) delivery to neuronal mitochondria. According to the results, MA membranes endowed RVG/TPP‐MASLNs with less reticuloendothelial system clearance, and reduced immunorecognition by inheriting the natural ability of macrophages and could not only efficiently penetrate BBB but also target the neuron cells and further localized in the mitochondria. This also demonstrates that ROS clearance via neuronal mitochondria‐targeted delivery by the designed biomimetic nanosystems could be a powerful method for delaying the progression of AD.^[^ ^106^, ^344^ ^]^

In addition to the elimination of excessive ROS, suppressing neuroinflammation is another method to alleviate AD or reduce its symptoms.^[^ ^345^ ^]^ Immune recognition of excessive neurotoxins by microglia is a trigger for the onset of neuroinflammation in the brain, therefore, blocking active recognition of microglia while removing neurotoxins holds promise for fundamentally alleviating neurotoxin‐induced immune responses, but is very challenging.^[^ ^346^, ^347^ ^]^ In light of this, Cheng et al. developed a macrophage‐biomimetic versatile nanomedicine (OT‐Lipo@M) for inflammation‐targeted therapy against AD, aiming at neurotoxin neutralization and immune recognition.^[^ ^106^ ^]^ The loaded OT can be slowly released to downregulate the expression of immune recognition site Toll‐like receptor 4 (TLR4) on microglia, inhibiting the TLR4‐mediated proinflammatory signaling cascade. Intranasal administration of OT‐Lipo@M was adopted to directly target the brain by bypassing the BBB. Benefiting from the immunosuppressive strategy, OT‐Lipo@M exhibits outstanding therapeutic effects on ameliorating cognitive deficits, inhibiting neuronal apoptosis, and enhancing synaptic plasticity in AD mice.^[^ ^106^ ^]^ Other types of cells, such as stem cells have also been used as membrane sources for the development of CNP‐based cancer therapeutics.^[^ ^108^ ^]^ Recently, Huang et al. designed neural stem cell (NSC) membrane‐coated nanoformulation, RVG‐NV‐NPs, for AD therapy. NSC was coated on AgAuSe quantum dots.^[^ ^108^ ^]^ The NSC membrane was modified to overexpress RVG, targeting cerebrovascular ECs and nerve cells in the brain. This RVG‐NV‐NPs system effectively passed through BBB and targeted to nerve cells. Additionally, it incorporated NIR‐II QDs to monitor in vivo drug delivery. Fluorescence imaging of AD mouse brain tissue from AD mouse revealed the RVG‐NV‐NPs group showed abundant fluorescence in the brain tissue. This research highlights the exceptional BBB penetration and nerve cell targeting capabilities of the NSC membrane‐coated NPs system, offering a promising avenue for AD therapy.

#### 3.6.2 Metal–Organic Frameworks

MOFs are a class of porous materials formed by the assembly of metal ions or metal clusters with organic ligands. Due to their unique structural and chemical properties, MOFs are garnering significant interest as a promising class of materials for AD therapy and diagnosis. Their appeal lies in their remarkable characteristics. i) MOFs possess a substantial specific surface area and pore volume, which bestow upon them remarkable sorption capabilities. ii) MOFs demonstrate biocompatibility, rendering them suitable for medical applications without eliciting any harm or adverse reactions within the biological system. iii) MOFs can serve as efficacious imaging agents, facilitating the visualization and diagnosis of AD‐associated pathologies.^[^ ^348^, ^349^ ^]^

Various approaches have been suggested for leveraging MOFs in AD management.^[^ ^349^ ^]^ Such as exploiting the inherent metal and ligand properties of MOFs can facilitate the adsorption and removal of Aβ, inhibit Aβ aggregation, or serve effectively as drug delivery vehicles, offering precision with minimal side effects.^[^ ^350^, ^351^, ^352^, ^353^, ^354^, ^355^ ^]^

MOFs with customizable pore sizes and properties based on chosen metal ions and ligands have demonstrated the ability to adsorb various peptides and proteins.^[^ ^356^, ^357^ ^]^ Mensinger et al. initially studied the adsorption capacity of seven different MOFs (HKUST‐1, MIL‐53, MIL‐69, MIL‐88B, MIL‐100(Fe), MIL‐101(Cr)NDC, and UiO‐66) for Aβ peptides using gel electrophoresis.^[^ ^350^ ^]^ All MOFs, except for MIL‐100(Fe), exhibited complete Aβ adsorption from the solution. Particularly, MIL‐101(Cr)NDC, UiO‐66, and MIL‐88B demonstrated rapid adsorption rates, where Aβ levels went below the detection limits of both assays (silver staining and fluorometric analysis) within 10 min of incubation. This significantly broadens the range of MOFs known to interact with Aβ. Nonetheless, further research is required to understand the adsorption mechanisms and interactions between peptides and MOF surfaces.^[^ ^350^ ^]^

**Figure 27:**
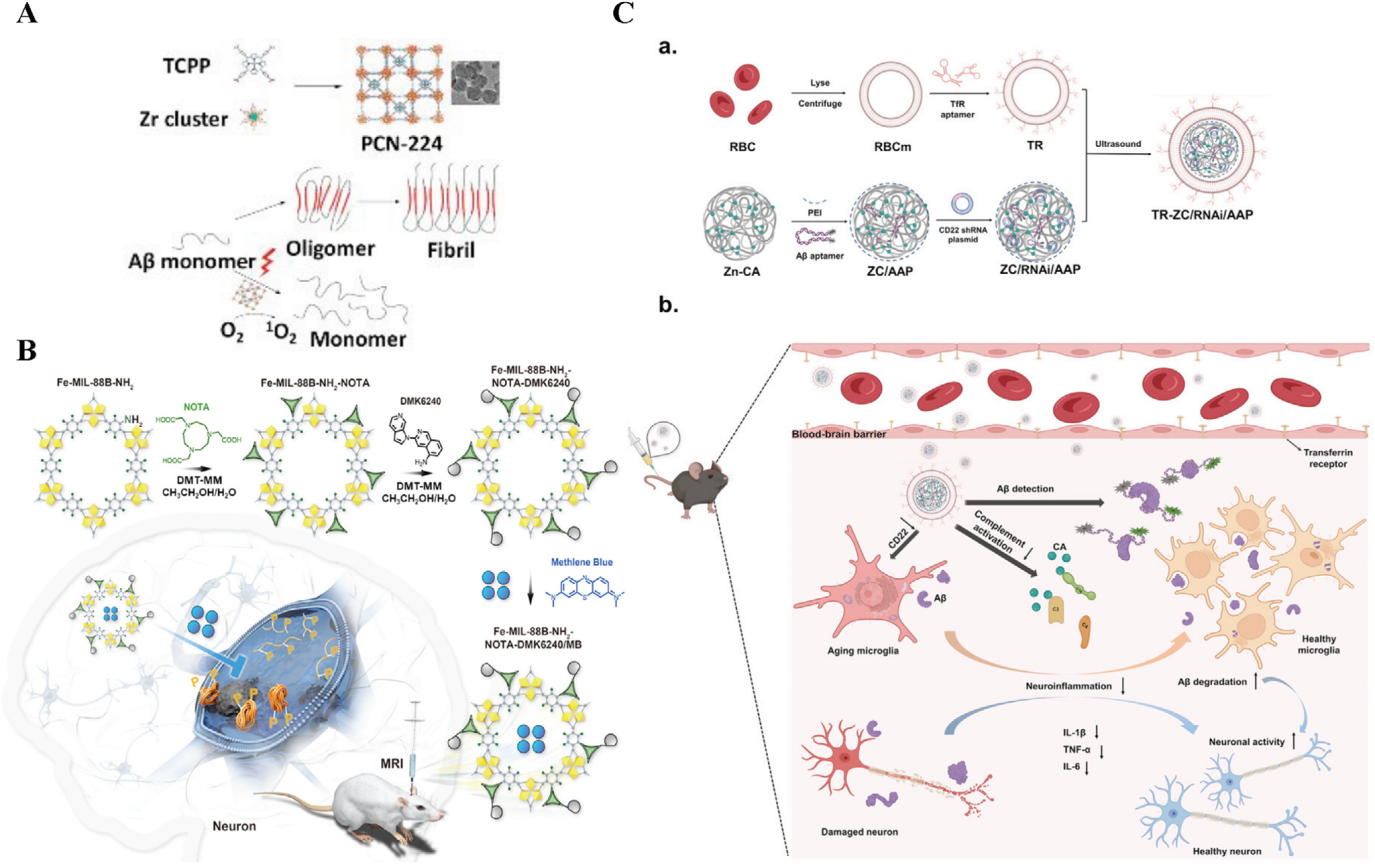
MOFs for AD treatment. A) Schematic diagram of the photoinhibition of Aβ_42_ aggregation by PCN‐224 nanoparticles. Reproduced with permission.^[^ ^358^ ^]^ Copyright 2018, American Chemical Society. B) Preparation and application of Fe‐MIL‐88B‐NH_2_‐NOTA‐DMK6240/MB. Reproduced with permission.^[^ ^355^ ^]^ Copyright 2020, American Chemical Society. C) Schematic illustration of TR‐ZC/RNAi/AAP preparation and its regulation for AD therapy. Reproduced under the terms of the Creative Commons Attribution 4.0 International Public License.^[^ ^359^ ^]^ Copyright 2020, Xinmin Nie et al.

In addition to clearing Aβ via adsorption, another effective strategy for AD treatment is to inhibit Aβ aggregation through photooxidation.^[^ ^360^, ^361^, ^362^ ^]^ Researchers discovered that oxygenated Aβ monomers generally have a reduced propensity for spontaneous aggregation compared to native Aβ monomers.^[^ ^360^, ^361^, ^362^ ^]^ To inhibit Aβ aggregation, LPFFD‐modified Hf‐MOF^[^ ^351^ ^]^ and PCN‐224,^[^ ^358^ ^]^ based on the photooxidation approach, were developed. Wang et al. devised porphyrinic MOF, PCN‐224 nanoparticles, that achieved NIR‐mediated Aβ aggregation suppression (**Figure** **27**A).^[^ ^358^ ^]^ The light‐induced inhibition of Aβ_42_ can be attributed to the generation of ROS by photoactivated PCN‐224 nanoparticles resulting from porphyrin as an effective photosensitizer for singlet oxygen (^1^O_2_) production. Therefore, PCN‐224 transformed harmful Aβ into less aggressive oxygenated forms via photooxygenation. Due to the intrinsic porosity of MOFs, PCN‐224 promoted photoinduced ^1^O_2_ generation, enhanced cell viability and reduced Aβ_42_‐induced toxicity, underscoring their potential in mitigating AD‐related cytotoxicity by inhibiting Aβ aggregation.^[^ ^358^ ^]^

Another prevalent MOF‐based method for treating AD leverages the adjustable morphology and surface chemistry of MOFs. MOFs serve as carriers to maintain drug stability, regulate drug release, and enhance transportation efficiency. UiO‐66, for example, has been employed as a vehicle for magnolol delivery (Mag@UiO‐66(Zr)), enhancing magnolol's water solubility and bioavailability.^[^ ^352^ ^]^ Magnolol has been shown to reduce β‐secretase 1 (BACE1) expression, which in turn leads to decreased Aβ plaque formation. Results indicated that compared to free‐form magnolol, MOF‐based nanoparticles exhibited superior β‐secretase inhibitory activity, and displayed enhanced neuroprotective activities, including reduced apoptotic neuron and neutrophil infiltration.^[^ ^352^ ^]^ Recently, Yu et al. constructed a zeolitic imidazolate framework‐8 (ZIF‐8) multifunctional drug delivery system for Aβ oligomer imaging and chemo‐photothermal treatment in living cells.^[^ ^353^ ^]^

Some strategies exploit MOF nanoparticle features, integrating diagnostic and therapeutic functions into a single nanoplatform.^[^ ^108^, ^354^, ^355^ ^]^ For instance, the magnetic iron‐based MOF material (Fe‐MIL‐88B‐NH_2_) functionalized with Tau‐targeting agent DMK6240 and Tau aggregation inhibitor methylene blue, was constructed as a Tau‐specific delivery platform (Fe‐MIL‐88B‐NH_2_‐NOTA‐DMK6240/MB) as shown in Figure 27B.^[^ ^355^ ^]^ Due to its rich iron content, this nanocomposite was found to be an excellent MRI contrast agent. The platform not only effectively protected tauopathy cells from death but also improved learning and memory capability, highlighting the theragnostic potential of MOF‐based nanoplatforms.^[^ ^355^ ^]^

Su et al. harnessed MOFs’ multiple voids and high drug‐loading capabilities, to load CD22shRNA plasmid for targeted immune regulation and Aβ aptamer for Aβ detection, constructing a Zn‐CA MOF as shown in Figure 27C.^[^ ^359^ ^]^ To achieve effective treatment by crossing the BBB, they coated MOF with an erythrocyte membrane and modified it with TR receptor aptamers. Results demonstrated that their Zn‐CA nanoparticles showed successful penetration into the brain with prolonged circulation half‐life, reduced recognition and enhanced clearance by the immune system.^[^ ^117^ ^]^

Although research on the application of MOFs for AD treatment is still in its infancy, the existing studies evidence the potential of MOFs as versatile nanomaterials capable of performing a variety of functions. The research on MOFs for treating other brain disorders, such as glioma, Parkinson's disease, and cerebral ischemic, is also noteworthy and instructive.^[^ ^363^, ^364^, ^365^, ^366^ ^]^ These conditions all share the common challenge of drug delivery across the BBB and some also have similar therapeutic pathways, such as alleviating neuroinflammation and reducing ROS, etc.^[^ ^352^, ^365^, ^366^ ^]^ While MOFs, with their unique properties, have opened avenues for drug delivery, challenges persist, particularly in navigating the complexities of the BBB and the need for further optimization in shape, size, surface chemistry, and composition to cater to personalized treatments and diagnostics. Beyond their traditional role as drug carriers, MOFs also hold vast opportunities in drug‐free therapeutic approaches, such as photothermal therapy and sonodynamic therapy, potentially broadening the treatment horizons for AD and other brain diseases. Integrated MOF‐based nanoplatforms present a promising pathway forward, hinting at the convergence of enhanced therapeutic efficacy, neural imaging, and improved bioavailability.

### 3.7 In Vitro Diagnosis of Novel AD Biomarkers

Mounting number of research has brought significant attention to the value of miRNA as biomarkers since miRNAs are key regulators in gene expression and are often implicated in the abnormal growth or function of certain cells, such is the case for neurodegenerative diseases.^[^ ^367^ ^]^ Although most of the initial findings highlighting diagnostic potential of miRNAs were in the field of oncology, significant work has recently been published endeavoring to investigate the role of miRNAs in different neurodegeneration diseases. Their role is not confined to merely a diagnostic viewpoint, but also provides a further understanding of disease etiology, making them increasingly essential in the relevant disease management and cure.

Several miRNA profiling experiments have identified and validated AD‐specific miRNAs. For example, hsa‐miR‐106, hsa‐miR‐153, and hsa‐miR‐101 have been shown to target APP while hsa‐miRNA‐29 and hsa‐miR‐107 have been shown to target BACE1, linking them to regulation of amyloid production in AD brains.^[^ ^368^ ^]^ Much attention has been focused on these miRNAs to determine if differential levels are found in circulating fluids, including blood and CSF. Hsa‐miR‐29a/b was well elaborated and proved intertwined with AD brain pathology, which is downregulated in serum of AD patients.^[^ ^369^ ^]^ Other examples of particular interest are expression patterns of hsa‐miR‐9 and hsa‐miR‐132, which were relevant to the alterations in stem cell commitment, neuronal differentiation, and actin remodeling. Their role in the disease could be mediated through the regulation of key pathways, such as axon guidance, longevity, insulin, and MAPK signaling pathway.^[^ ^370^ ^]^

Monitoring miRNA dysregulation can be used to elucidate the details of AD processes relevant to disease pathogenesis to a greater extent. In this case, the pathology and mechanisms of AD, along with the dysregulation of miRNA, are able to be leveraged as effective indicators for the presence of the disease. The benefits of using miRNAs as biomarkers for AD diagnosis have been manifest and apparent: apart from diversified expression under different disease stages, miRNAs are also easily accessible and allow noninvasive sample collection due to their presence and high stability in biofluids such as blood, urine, and saliva. By combining with nanomaterials as carriers to immobilize or carry miRNA‐related detection agents, miRNAs are relatively easy to work with and assay in vitro, exhibiting remarkable and tremendous promising future in efficient, facile yet precise biomarker‐based detection of miRNAs, therefore the diagnosis of AD or other neurodegenerative disorder ultimately.

## 4 Conclusion

AD as a neurodegenerative disorder still remains no definitive cure to date. While a multitude of inspiring progress has been made in AD diagnosis and treatment. Conventional drug therapies primarily focus on symptom alleviation but are often accompanied by notable side effects. In recent decades, nanotechnology has garnered widespread attention, offering new prospects for the treatment and diagnosis of AD. Nanomaterials, owing to their nanoscale properties, enable precise engineering at the molecular level to enhance their in vivo biodistribution, drug release, and cellular targeting. Furthermore, nanomaterials can be employed to develop high‐resolution imaging techniques for early diagnosis of AD. Researchers have successfully developed various nanomaterials for drug delivery and imaging, which not only capable of traversing the BBB, but also serve multiple therapeutic and diagnostic functions.

However, despite the immense potential of nanotechnology in AD research, challenges still persist, among which, the primary challenge is to overcome the BBB efficiently. Although various strategies such as RMT and external forces have been proposed, they still encounter limitations and safety concerns. RMT may face issues like receptor saturation and immune rejection, while the application of external forces could involve complex equipment and safety issues. Furthermore, most nanomaterials exhibit limited in vivo biodistribution and circulation time, preventing prolonged presence in the brain thus demonstrating inadequate therapeutic effects. To this end, focus can be turned to facile noninvasive biomarker‐based precise detection of AD, which has gained increasing interest as in vitro diagnosis strategy that occur in body fluids like blood or urine, minimizing the pain otherwise caused to disease‐stricken patients. Alternatively, on top of in vitro diagnosis, future research and development of nanomaterials for in vivo AD treatment or diagnosis can be focused on the following directions.

- Developing innovative BBB crossing strategies: novel approaches to overcome the formidable BBB can be further explored, which may include developing more precise RMT to ensure efficient NP traversal, circumventing the current issues stemming from receptor saturation or immune rejection. Additionally, further investigation into the application of external forces could determine their potential to assist nanoparticle passage through the BBB.
- Prolonging circulation duration: concentration can be made on designing and improving nanomaterials to extend their circulation time in vivo, which can be achieved through enhancements in surface modification, coating materials, and NP size control. Cell membrane‐coated nanoparticles show promise in this regard as the coated membranes significantly extend the retention time of NPs. Prolonged circulation time could also increase the chances of NPs entering the brain. Additionally, considering the combination of nanotechnology with other treatment methods, such as immunotherapy or gene therapy, could be promising in boosting the therapeutic efficacy. It can be deduced that the combination of different treatment modalities can lessen the reliance on prolonged presence of NPs in the brain.
- Conducting long‐term toxicity studies: greater emphasis is supposed to be placed on the long‐term toxicity of nanomaterials, including their accumulation, impact on the nervous system, and potential harm, which entails studying pharmacokinetics, metabolism, clearance, tissue accumulation, biodegradation, and immune system effects. Moreover, apposite consideration could be given to developing methods for NPs retrieval and recycling. Taking measures to retrieve NPs once they have completed their therapeutic or imaging tasks could mitigate dependence on prolonged biodistribution and circulation time, forestalling latent harm associated with their in vivo presence.
- Further developing multifunctional nanosystems: future research can focus on the development of broader multifunctional nanomaterials that are rendered capable of crossing the BBB and serving multiple therapeutic and diagnostic functions.

Integrated diagnosis and treatment: devising intelligent nanomaterials that integrate diagnosis and treatment functionalities on a single platform. By carrying various drug carriers and contrast agents, theranostic nanomaterials not merely provide precise diagnosis but also offer targeted therapy, striving to harness the versatility of nanomaterials to the fullest.

Multifunctional therapy: further improving NPs to achieve multiple therapeutic functions, including anti‐inflammatory effects, alleviation of oxidative stress, and enhancement of mitochondrial function. Such multifunctionality can provide comprehensive treatment targeting multiple pathological features of AD.

Smart control: there is still room for vaster types of intelligent control systems for NPs that respond to specific environmental conditions inside the brain. For instance, NPs could release drugs upon encountering the stimuli from neuroinflammation or oxidative stress in brain, ensuring precise treatment.

Making breakthroughs in early diagnosis and prevention strategies: in addition to treatment, early diagnosis and prevention remain critical when it comes to AD. Future research can focus on developing earlier diagnostic methods, potentially based on biomarkers or imaging techniques.

To conclude, future research efforts could be dedicated to addressing challenges related to BBB traversal, long‐term toxicity, multifunctional nanomaterials, and early diagnosis, all of which will pave the way for more effective treatments and earlier diagnoses for AD. Achieving these goals will require multidisciplinary collaboration and continuous innovation to make breakthroughs in the field of AD research.

## Conflict of Interest

The authors declare no conflict of interest.
